# Supplementary material for: Incorporating evolutionary and threat processes into crop wild relatives conservation
Source: Nat Commun. 2022 Oct 21;13:6254. doi: 10.1038/s41467-022-33703-0 (PMC9587227; doi:10.1038/s41467-022-33703-0)
Supplement: Supplementary file 1 — Supplementary Information [file 41467_2022_33703_MOESM1_ESM.pdf]

Supplementary Information

Incorporating evolutionary and threat processes into crop wild relatives conservation

Tobón-Niedfeldt W., Mastretta-Yanes A., Urquiza-Haas T., et al.

Supplementary Figures

Supplementary Figure 1 2
Supplementary Figure 2 3
Supplementary Figure 3 4
Supplementary Figure 4 5
Supplementary Figure 5 14
Supplementary Figure 6 15
Supplementary Figure 7 16
Supplementary Figure 8 17
Supplementary Figure 9 18
Supplementary Figure 10 19
Supplementary Figure 11 20
Supplementary Figure 12 21
Supplementary Figure 13 22
Supplementary Figure 14 23
Supplementary Figure 15 24

Supplementary Notes

Supplementary Note 1 25
Supplementary Note 2 25
Supplementary Note 3 25
Supplementary Note 4 26
Supplementary Note 5 26
Supplementary Note 6 27

Supplementary References 28

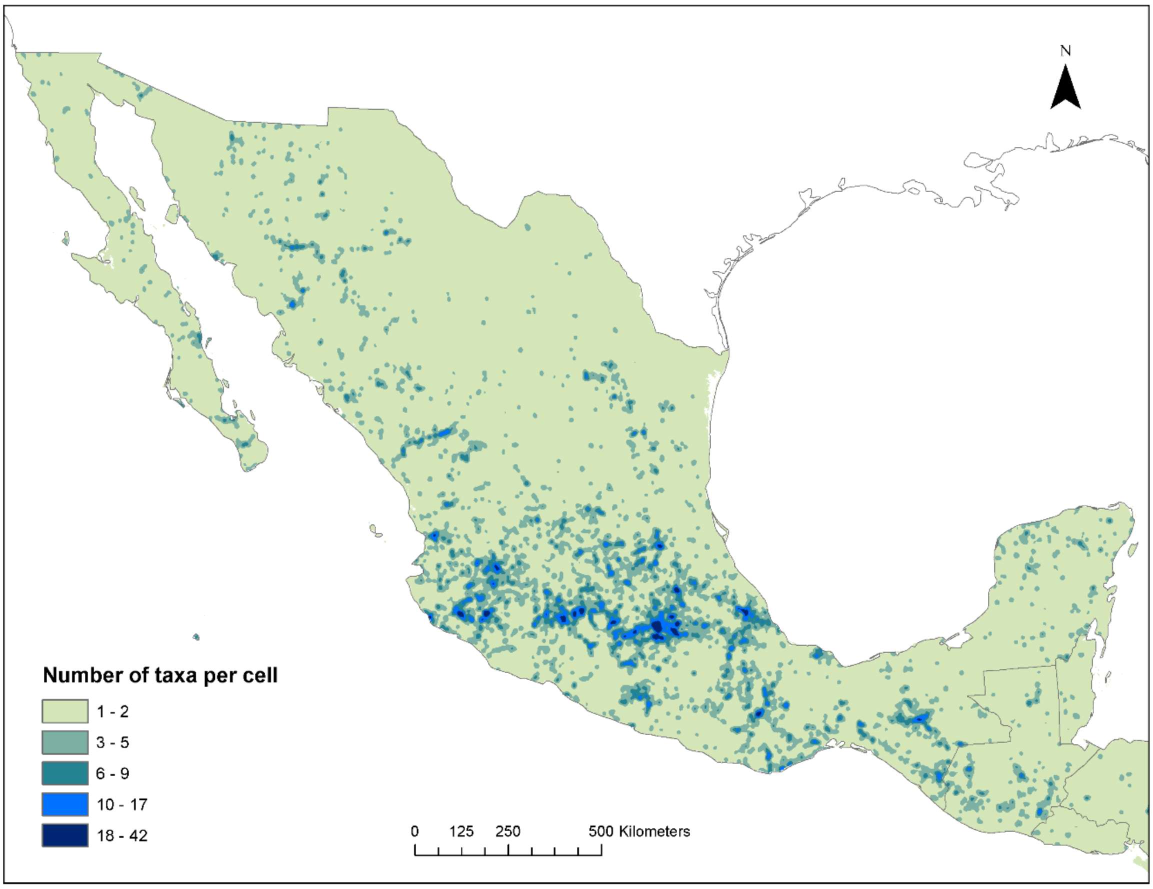

Supplementary Figure 1. Spatial pattern of taxa richness of selected Mesoamerican crop wild relatives in Mexico based on occurrence georeferenced data. Spatial resolution 5 km^2. [Country boundaries according to Natural Earth.]

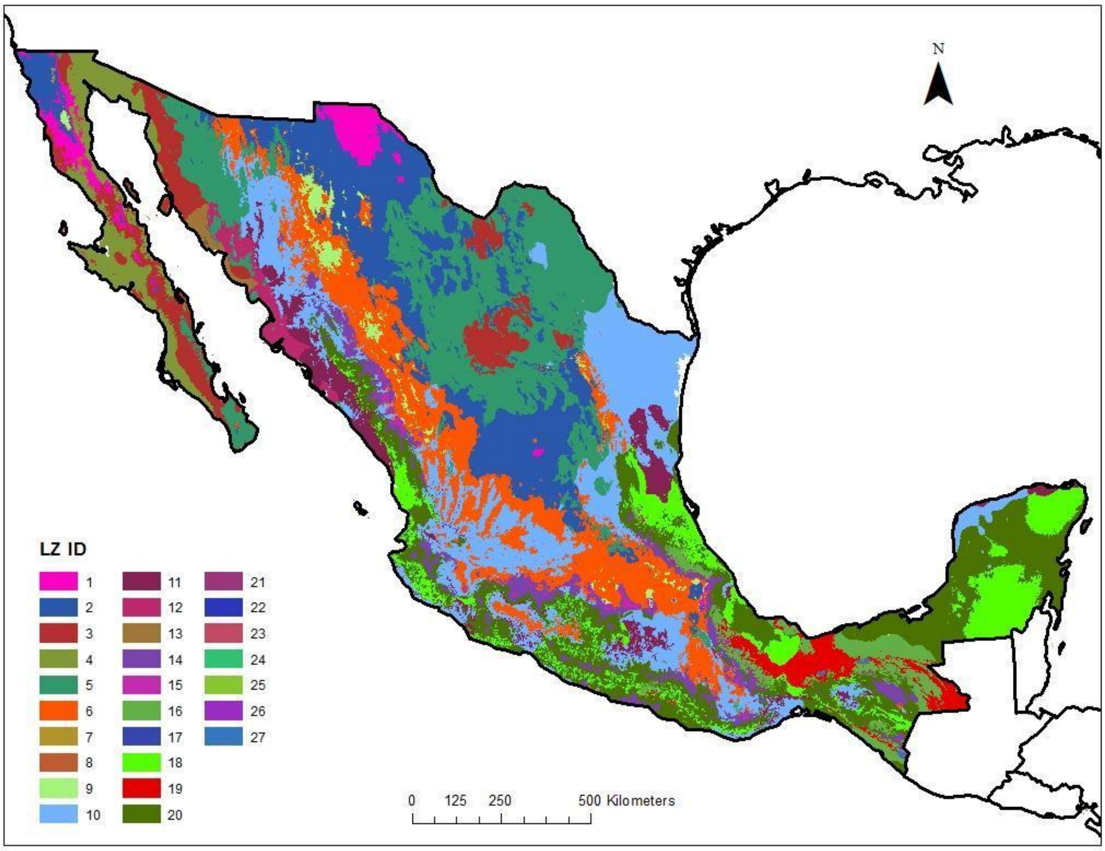

Supplementary Figure 2. Holdridge life zones (LZ) of Mexico, based on bio-temperature, annual precipitation and potential evapotranspiration ratio (see unique identifiers (ID) Supplementary Data 8). [Country boundaries according to Natural Earth.]

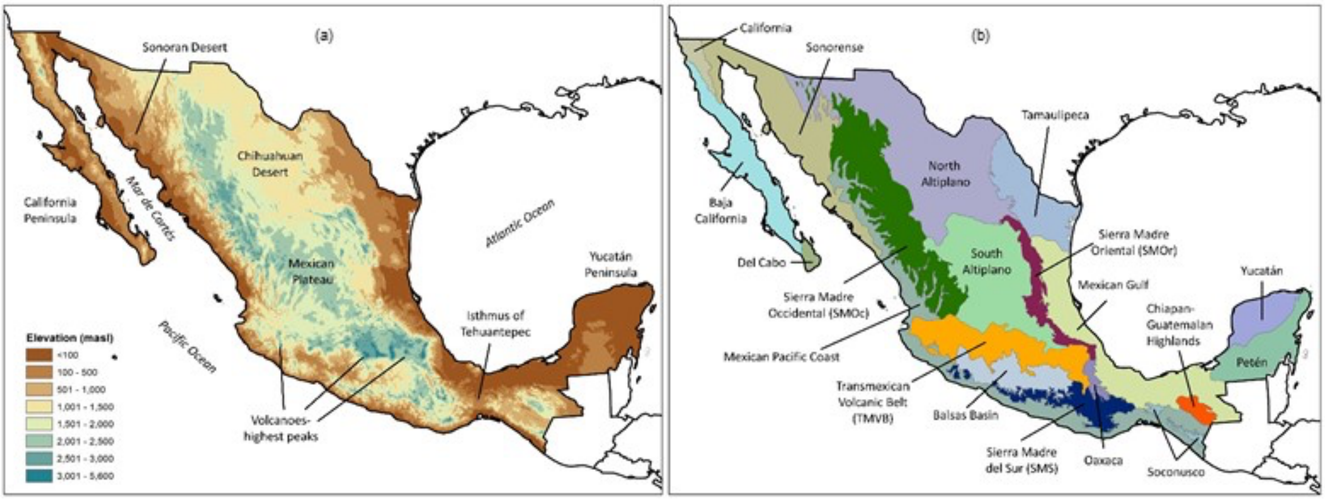

Supplementary Figure 3. Elevation[1], indicating main geographic references (panel a), and biogeographic provinces[2] of Mexico (panel b). [Spatial data is licensed under CC-BY 2.5; country boundaries according to Natural Earth.]

Life Zone (LZ)	Number of PGD after division	Rationale
LZ 1 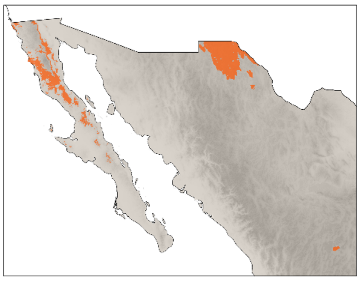	3 PGD 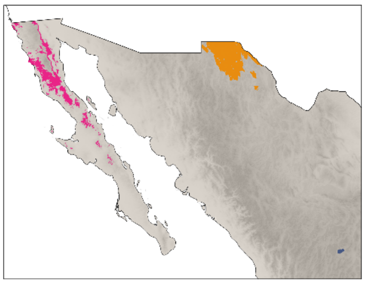	Polygons are isolated by the SMOc and Sonoran Desert. Polygon to the South is very isolated within the South Altiplano. Arid taxa of these regions have been found to be genetically differentiated.<br><br>References: [3-6]<br><br>Division based on: Biogeographic provinces
LZ 2 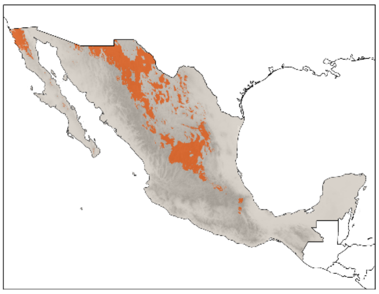	4 PGD 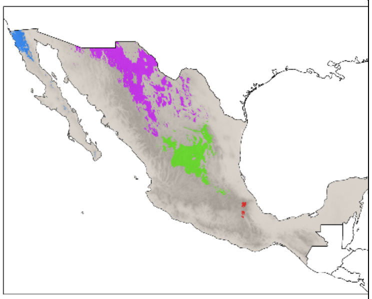	California Peninsula is isolated by the Sonoran Desert. A North/South pattern of genetic differentiation has been found in the Altiplano. Other areas to the South are isolated by the TMVB. Arid taxa of these regions have been found to be genetically differentiated.<br><br>References: [4-8]<br><br>Division based on: Biogeographic provinces
LZ 3 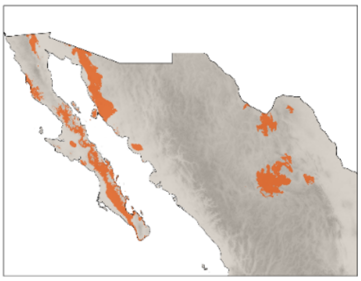	3 PGD 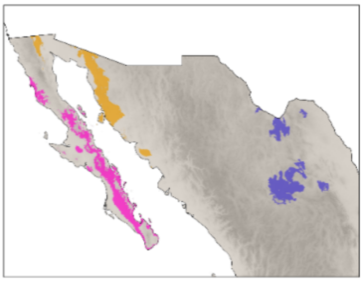	Taxa from the California Peninsula and the Sonoran Desert have been found to be differentiated due to the orogenic history of the Peninsula. Polygons to the East are separated from the Sonoran Desert by the SMOc.<br><br>References: [3,6,9–11]<br><br>Division based on: Biogeographic provinces
LZ 4	2 PGD	A mountain range divides the two lowland areas, one to the Mar de Cortés and another to the Pacific Ocean, among which coastal taxa have been found to be differentiated.<br><br>References: [3,12,13]

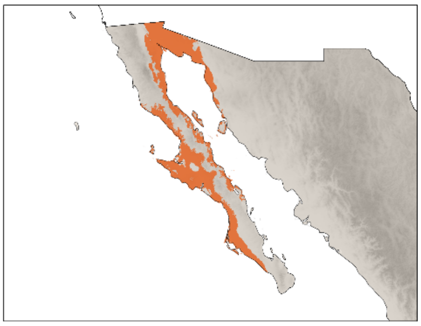	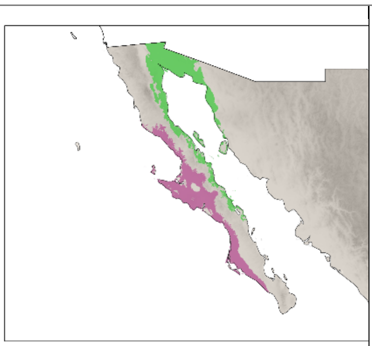	Division based on: Topography (mountain chain)
LZ 5:<br>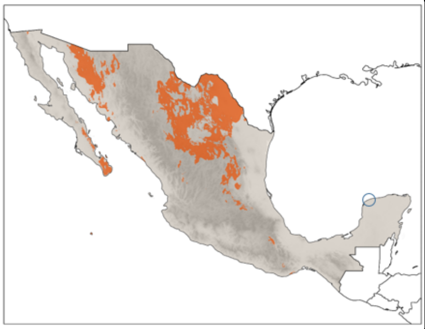	6 PGD<br>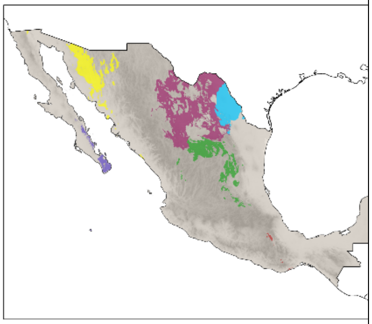	Californian Peninsula is isolated by the Mar de Cortés. The Sonoran and Chihuahuan Deserts are divided by the SMOc, which acts as a barrier. A North/South pattern of genetic and biogeographic differentiation has been found in the Altiplano and in populations to the East (Tamaulipeca province).<br><br>References: [3,7–9,14,15]<br><br>Division based on: Biogeographic provinces<br><br>Note: Small area in Yucatán Peninsula were fused with LZ 10.
LZ 6: 546<br>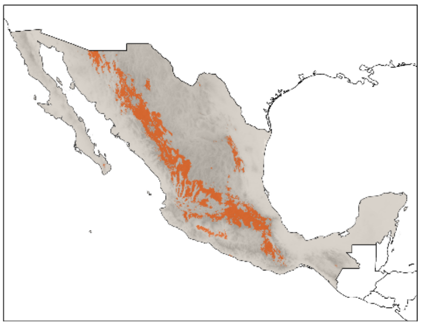	11 PGD<br>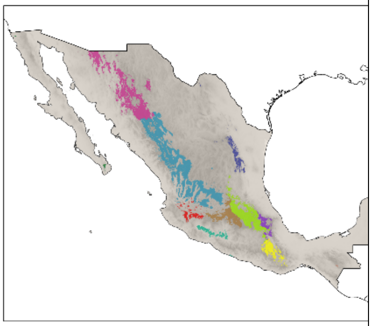	SMOc, SMOr, TMVB and SMS highlands have different geologic ages and are separated by lowlands, leading to population differentiation of highland taxa. There is a North/South phylogeographic break within the SMOc likely related to differential range shifts during the Pleistocene. Population differentiation has been found among different parts of the TMVB, likely related to its geologic history and not to current topography.<br><br>References: [16–32]<br><br>Division based on: Biogeographic provinces, basins, and topographic features as close as possible to known phylogeographic breaks.

LZ 7<br>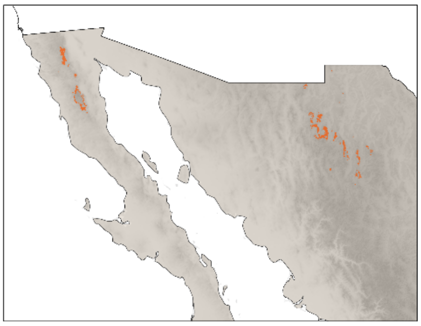	2 PGD<br>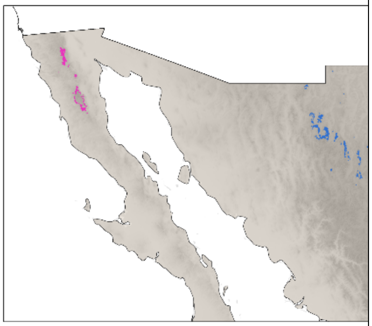	The Highlands of California Peninsula are isolated from the SMOc by the Mar de Cortes and Sonoran Desert. Sonoran Desert acts as a barrier.<br><br>References: [3-6]<br><br>Division based on: Biogeographic provinces
LZ 8<br>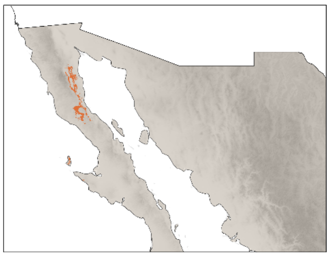	Not divided	LZ is too small to show general patterns of genetic differentiation.
LZ 9<br>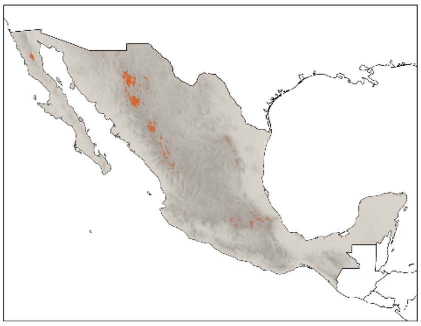	6 PGD<br>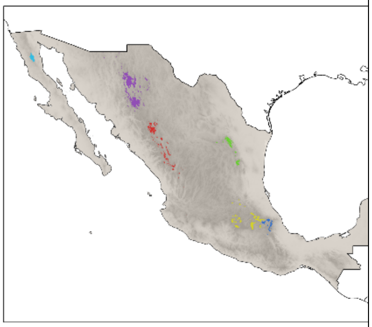	SMOc, SMOr, TMVB and SMS highlands have different geologic ages and are separated by lowlands, leading to population differentiation of highland taxa. There is a phylogeographic break North/South within the SMOc, likely related to differential range shifts during the Pleistocene. Population differentiation has been found among different parts of the TMVB, likely related to its geologic history and not to current topography.<br><br>References: [16–19,22,27,29–31,33]<br><br>Division based on: Biogeographic provinces, basins, and topographic features as close as possible to known phylogeographic breaks.

LZ 10<br>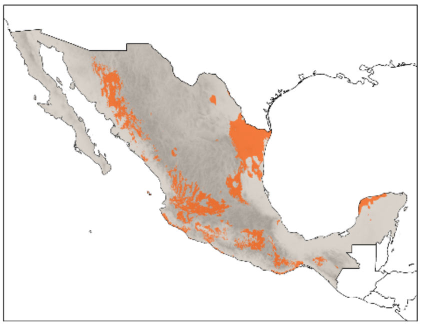	9 PGD<br>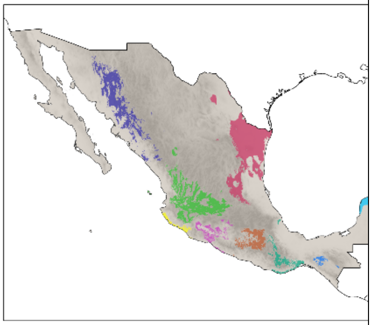	Coasts of the Pacific, Atlantic and Yucatan Peninsula are isolated by Mexico's mainland. Fragmented areas within the Balsas Basin, Pacific Coast and SMS presented climate stability during the Pleistocene climate fluctuations and currently hold differentiated populations.<br><br>References: [34–37]<br><br>Division based on: Biogeographic provinces
LZ 11<br>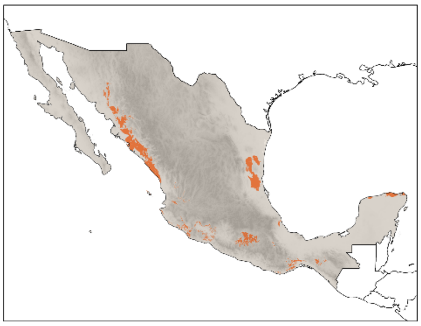	7 PGD<br>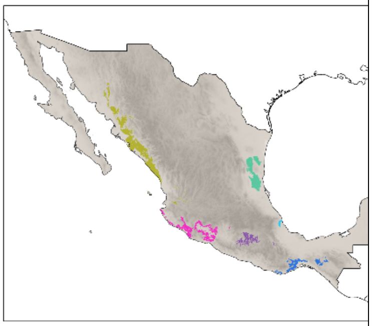	Coasts of the Pacific, Atlantic and Yucatan Peninsula are isolated by the mainland. Fragmented areas within the Balsas Basin, Pacific Coast and SMS presented climate stability during the Pleistocene climate fluctuations, and currently hold differentiated populations.<br><br>References: [38–40]<br><br>Division based on: Biogeographic provinces
LZ 12<br>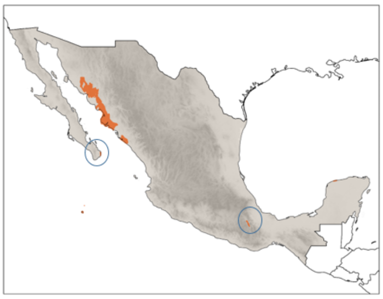	1 PGD<br>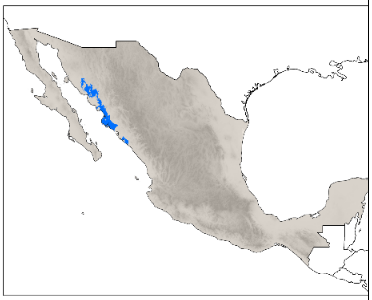	Not divided, but small isolated area in Del Cabo province and South Mexico were fused with LZ 5 and LZ 11.
LZ 13<br>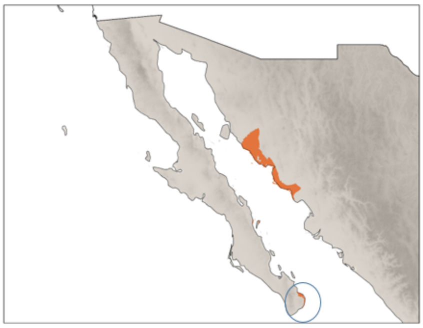	1 PGD<br>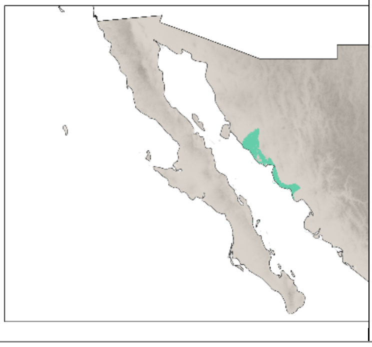	Not divided, but a small isolated area in Del Cabo province was fused with LZ 3.

LZ 14 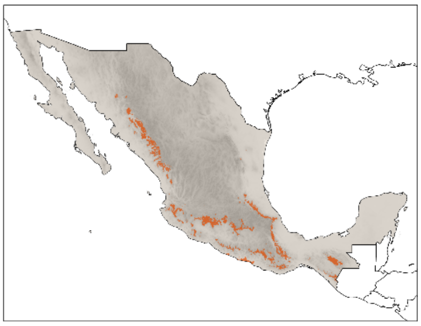	7 PGD 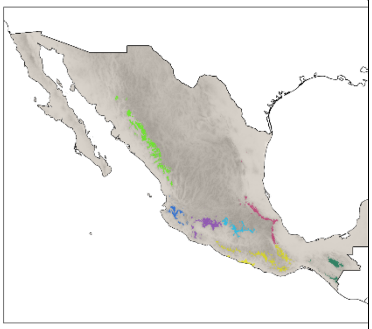	SMOc, SMOr, TMVB and SMS highlands have different geologic ages and are separated by lowlands, leading to population differentiation in highland taxa. Population differentiation has been found among different parts of the TMVB, likely related to its geologic history and not to current topography. The Isthmus of Tehuantepec has been identified as an important barrier for several taxa of different taxonomic groups.<br><br>References: [16–19,22,27,29–31,33,35]<br><br>Division based on: Biogeographic provinces, basins, and topographic features as close as possible to known phylogeographic breaks.
LZ 15 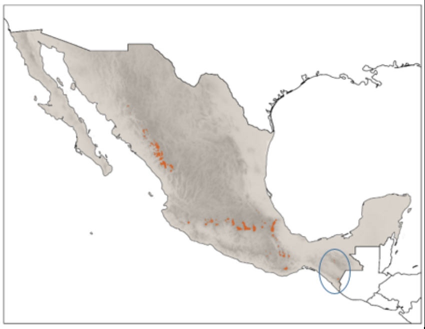	6 PGD 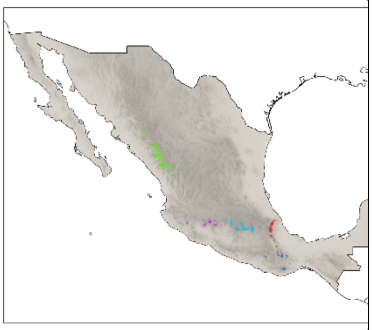	SMOc, SMOr, TMVB and SMS highlands have different geologic ages and are separated by lowlands, leading to population differentiation in highland taxa. Population differentiation has been found among different parts of the TMVB, likely related to its geologic history and not to current topography. Isthmus of Tehuantepec has been identified as an important barrier for several taxa of different taxonomic groups.<br><br>References: [16–19,27,30,33,35,41,42]<br><br>Division based on: Biogeographic provinces, basins and edaphic features as close as possible to known phylogeographic breaks.<br><br>Note: Small area in Chiapas was fused with LZ 14.
LZ 16	10 PGD	Fragmented areas within the Balsas Basin, Pacific Coast and SMS lowlands

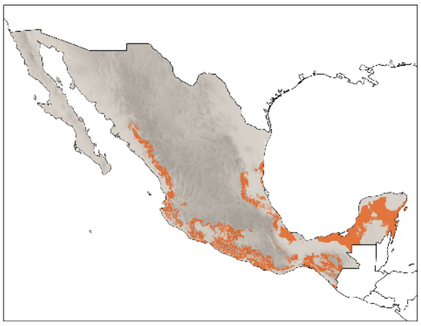	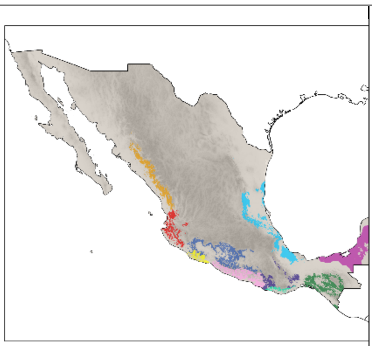	presented climate stability during the Pleistocene climate fluctuations, and currently hold differentiated populations. The Isthmus of Tehuantepec has been identified as an important barrier for several taxa of different taxonomic groups. Yucatán Peninsula is isolated from the Pacific Coast by the Chiapan-Guatemalan Highlands and from the Gulf of Mexico coast by the Isthmus of Tehuantepec.<br><br>References: [38,40,43–48]<br><br>Division based on: Biogeographic provinces and basins
LZ 17<br>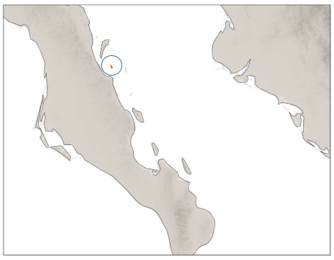	Not included in the analysis	LZ is only in a small island, which was excluded from the species distribution modelling and rest of analyses, as islands were not included in the assessment due to resolution issues.
LZ 18<br>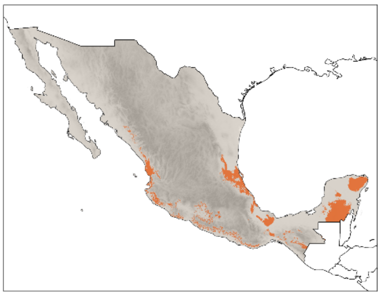	6 PGD<br>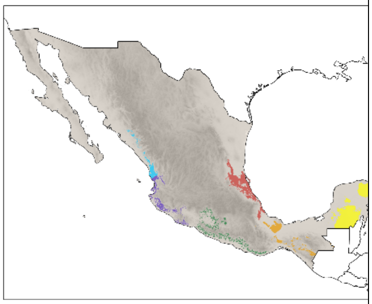	Fragmented areas within the Balsas Basin, Pacific Coast and SMS lowlands presented climate stability during the Pleistocene climate fluctuations, and currently hold differentiated populations. The Isthmus of Tehuantepec has been identified as an important barrier for several taxa of different taxonomic groups. Yucatán Peninsula is isolated from the Pacific Coast by the Chiapan-Guatemalan Highlands and by a climatic barrier.<br><br>References: [36–38,40,43,46,48–53]<br><br>Division based on: Biogeographic provinces and basins.
LZ 19	3 PGD	Chiapan-Guatemalan Highlands represent a barrier for the lowlands of

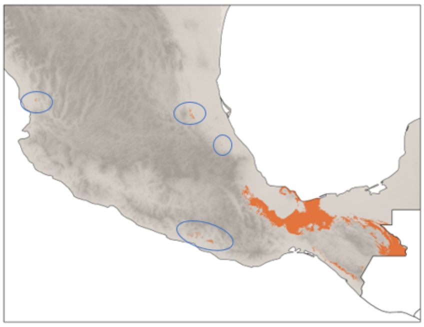	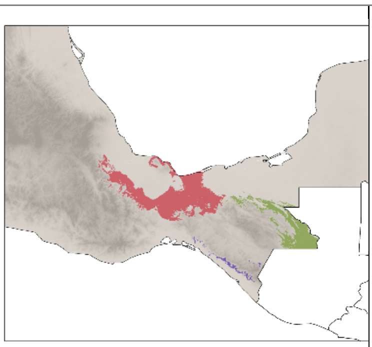	the Lacandona Jungle, the Pacific Coast and the Isthmus of Tehuantepec.<br><br>References: [35,40,43,44,54]<br><br>Division based on: Biogeographic provinces and basins.<br><br>Note: Small areas (blue circles) were fused with LZ 18.
LZ 20<br>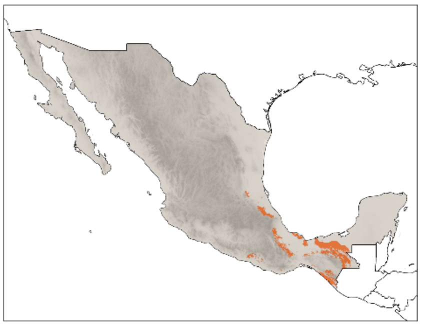	5 PGD<br>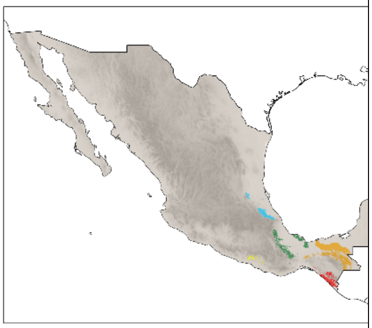	SMOr, SMS and Chiapan-Guatemalan Highlands have different geologic ages and are separated by lowlands, leading to population differentiation in highland taxa.<br>The Isthmus of Tehuantepec has been identified as an important barrier for several taxa of different taxonomic groups.<br><br>References: [35,53,55–58]<br><br>Division based on: Biogeographic provinces, basins, and topographic features as close as possible to known phylogeographic breaks.
LZ 21<br>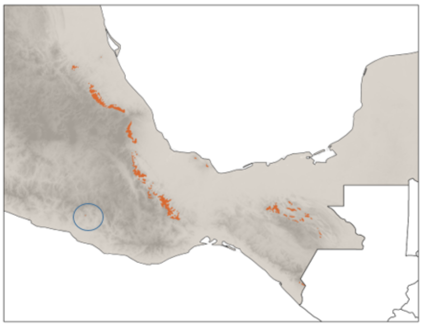	4 PGD<br>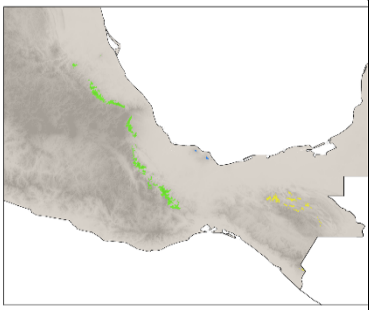	The Isthmus of Tehuantepec has been identified as an important barrier for several taxa of different taxonomic groups.<br><br>References: [53,55,56,59,60]<br><br>Division based on: Biogeographic provinces<br><br>Note: Small area to the West was fused with LZ 20.
LZ 22, 23, 24	2 PGD	These LZ represent the highest peaks of the volcanoes of the TMVB, where considerably low valleys separate them,; preventing glacial connectivity during the Pleistocene.

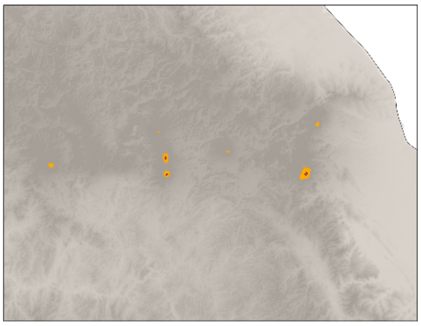	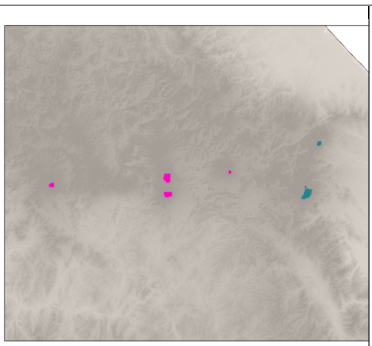	References: [27,33,61,62]<br><br>Division based on: Topography and basins<br><br>Note: LZ 22, 23 and 24 were very small, so they were first fused, and then divided.
LZ 25<br>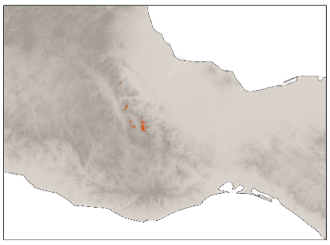	Not divided	LZ is too small to show general patterns of genetic differentiation
LZ 26, 27<br>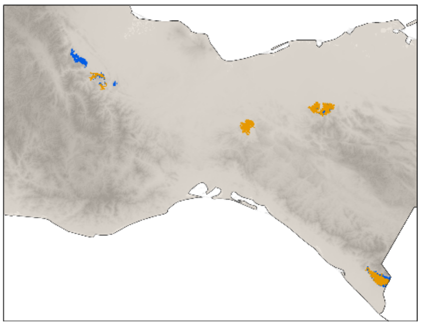	2 PGD<br>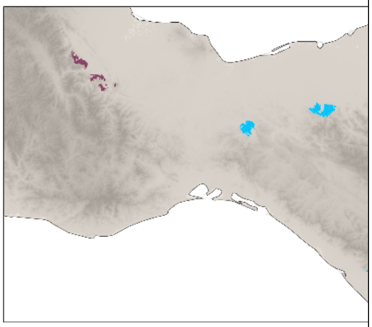	The Isthmus of Tehuantepec has been identified as an important barrier for several taxa of different taxonomic groups.<br><br>References: [56]<br><br>Division based on: Biogeographic provinces<br><br>Notes: LZ 26 and 27 were very small, so they were first fused, and then divided.

Supplementary Figure 4. Rationale for the division of each life zone (LZ) into proxies of genetic differentiation (PGD) based on the following cartography used for the division: biogeographic provinces[2], basins[63], topography[64], and edaphology[65]. (Abbreviations of biogeographic provinces as in Supplementary Figure 3. See references at the end of this document or in Supplementary Data 9.) [Spatial data is licensed under CC-BY 2.5; country boundaries according to Natural Earth.]

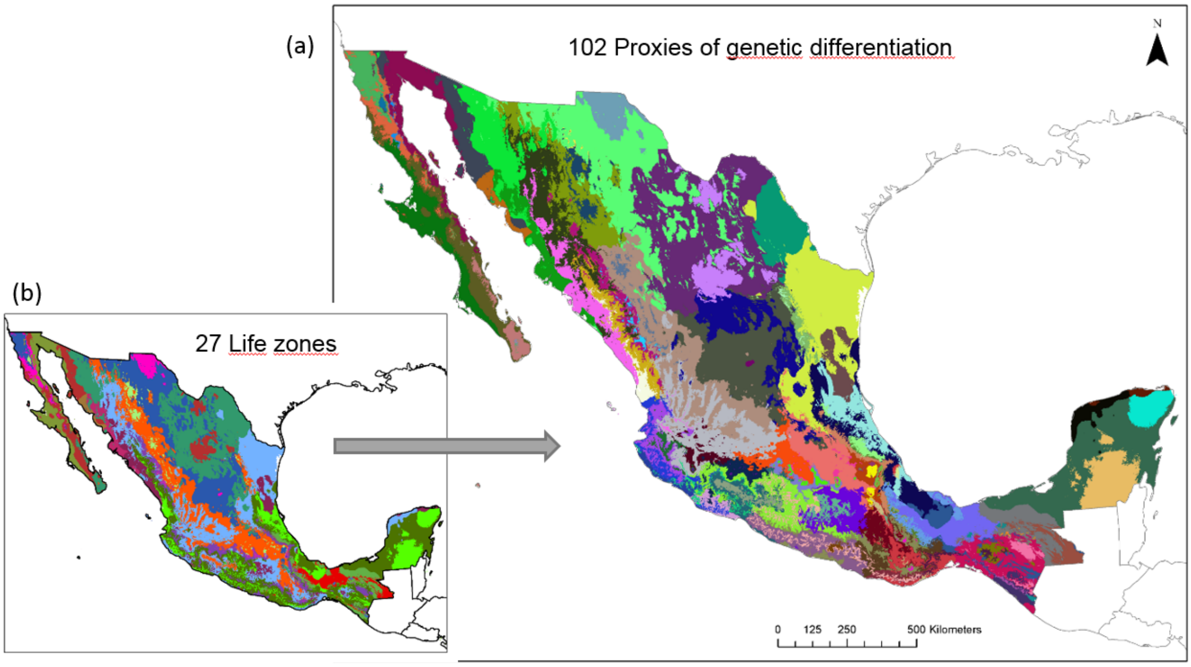

Supplementary Figure 5. Proxies of genetic differentiation for Mexico (panel a), based on environmental data as given by Holdridge life zones (see panel b or Supplementary Figure 2) and historic drivers as given by phylogeographic patterns. (See references at the end of this document or in Supplementary Data 9; see summary of taxonomic information in Supplementary Data 10; see division of each life zone into proxies in Supplementary Figure 4). [Country boundaries according to Natural Earth.]

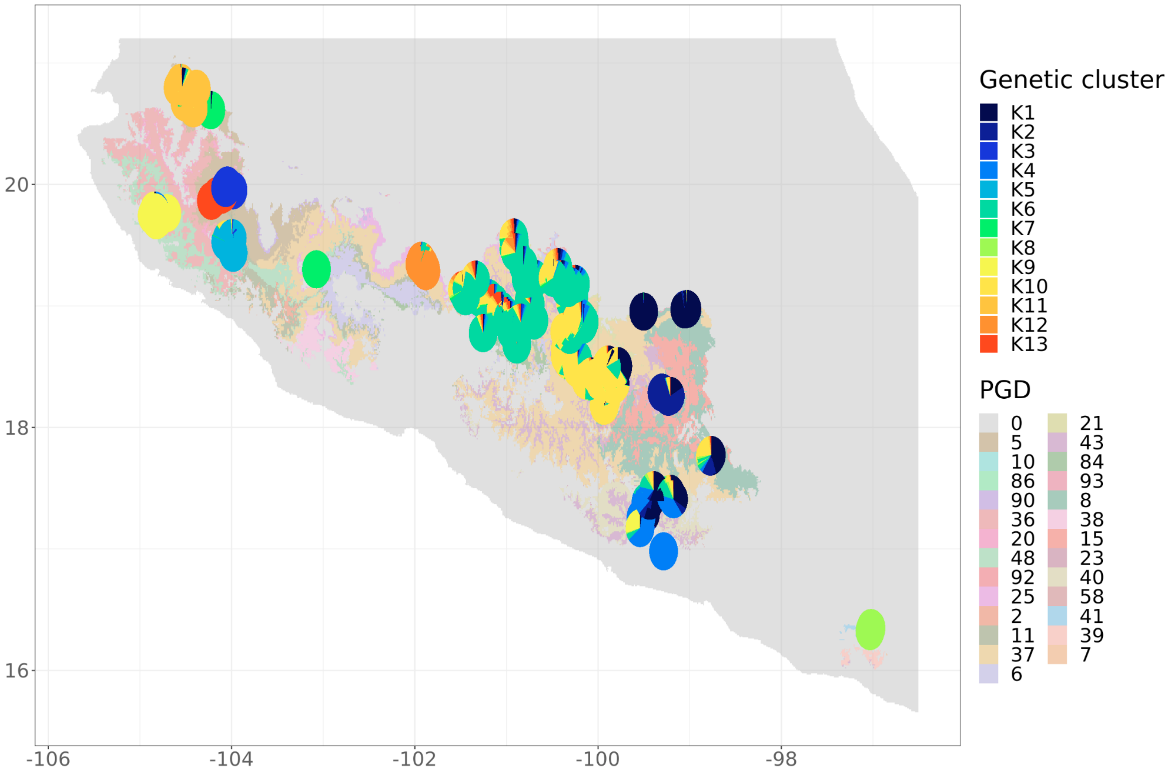

Supplementary Figure 6. Genetic diversity of *Zea mays* subsp. *parviglumis* represented in the proxies of genetic differentiation (PGD). Pies represent the proportion of Admixture clusters within sampling populations, assuming *K*=13, using around 30,000 SNPs (data from Rivera-Rodríguez[66]). The map shows the species distribution model of *Z. mays* subsp. *parviglumis* divided by proxies of genetic differentiation (background colors). [Spatial data is licensed under CC-BY 4.0; country boundary according to Natural Earth.]

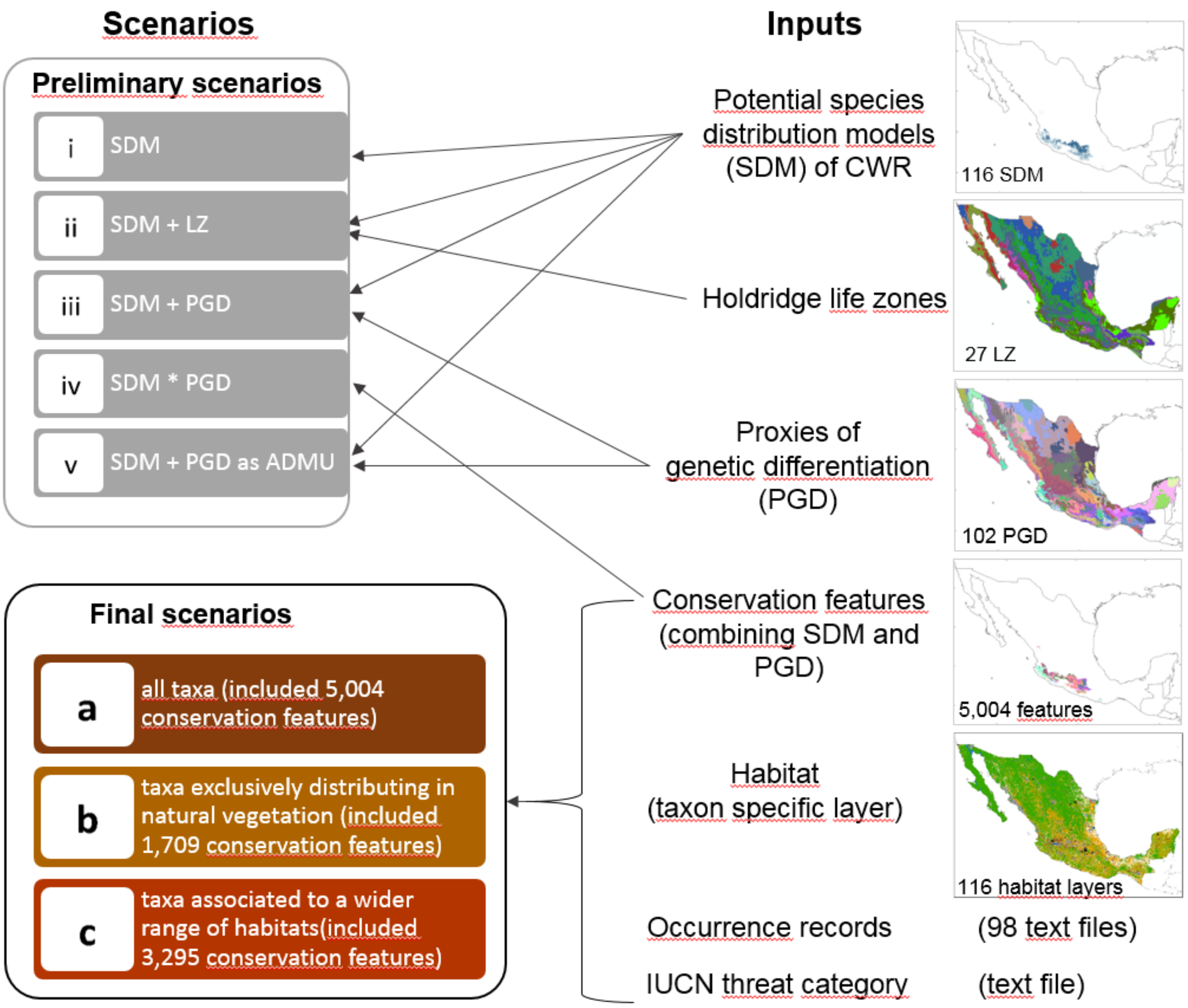

Supplementary Figure 7. Overview of the systematic conservation planning analysis performed with Zonation in the context of the present assessment to identify important areas for conservation of Mesoamerican CWR. (Abbreviations are as follows: SDM- Species distribution models; LZ - Holdridge life zones; PGD - Proxies of genetic differentiation; ADMU - Administrative Unit). [Spatial data is licensed under CC-BY 2.5 and 4.0; country boundaries according to Natural Earth.]

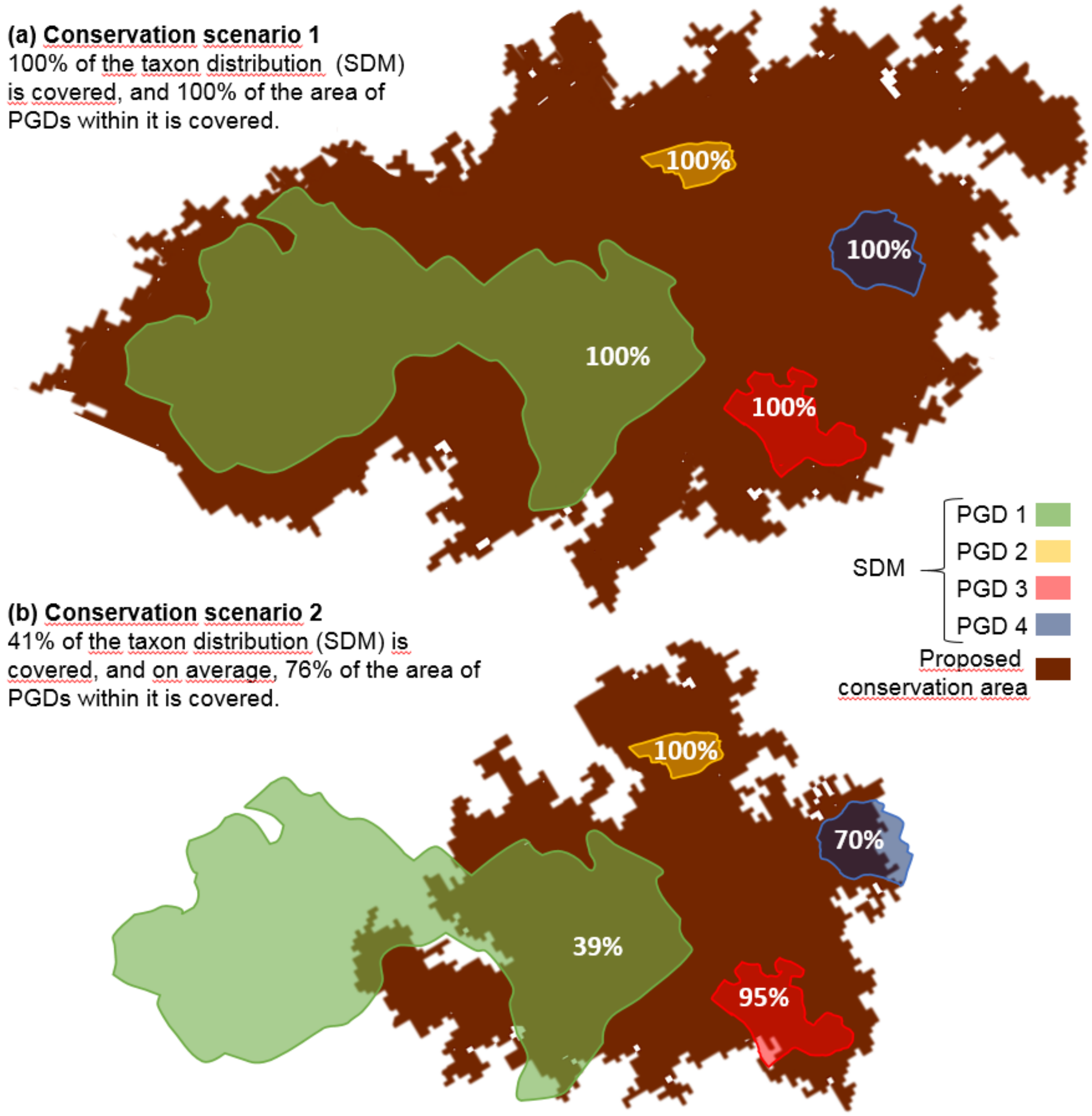

Supplementary Figure 8. Illustrated representation of the proxies of genetic differentiation (PGD) within a conservation area. A taxon, as given by a species distribution model (SDM) subdivided by four proxies of genetic differentiation, is overlaid on two scenarios of proposed conservation areas. (a) For scenario 1, all the areas of all proxies, and consequently the full taxon range as given by the SDM, are represented within the proposed conservation area. (b) Not all the areas of all proxies are represented within the proposed conservation area (e.g. only the 39% of the PGD 1 overlaps with the proposed conservation area), so on average, for this taxon 76% of the area of the PGDs are represented within the proposed conservation area. [Spatial data is licensed under CC-BY 2.5]

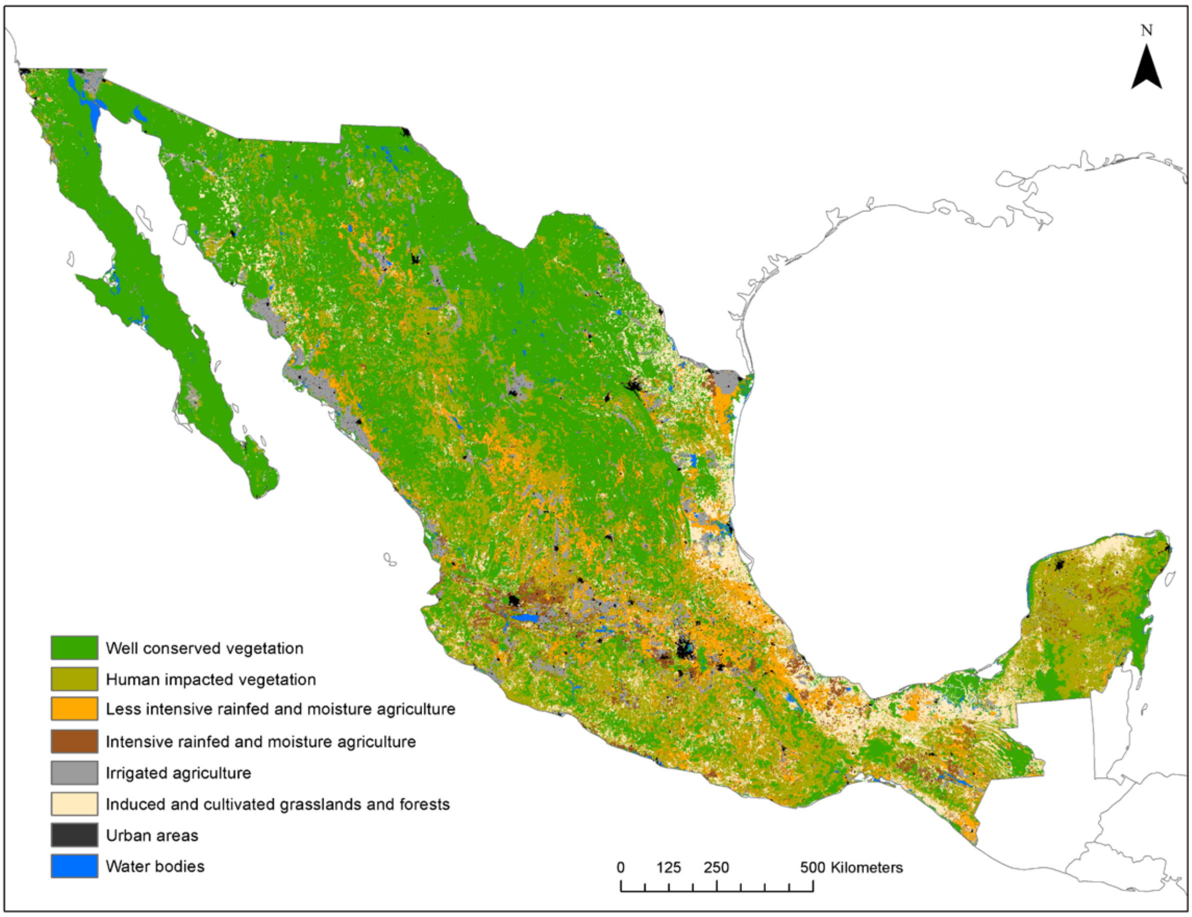

Supplementary Figure 9. Land cover map that was used to assess habitat preference of each taxon (see Supplementary Data 11 for taxon-specific data). Data was based on INEGI[67] and Bellon *et al.*[68]. [Spatial data is licensed under CC-BY 2.5; country boundaries according to Natural Earth.]

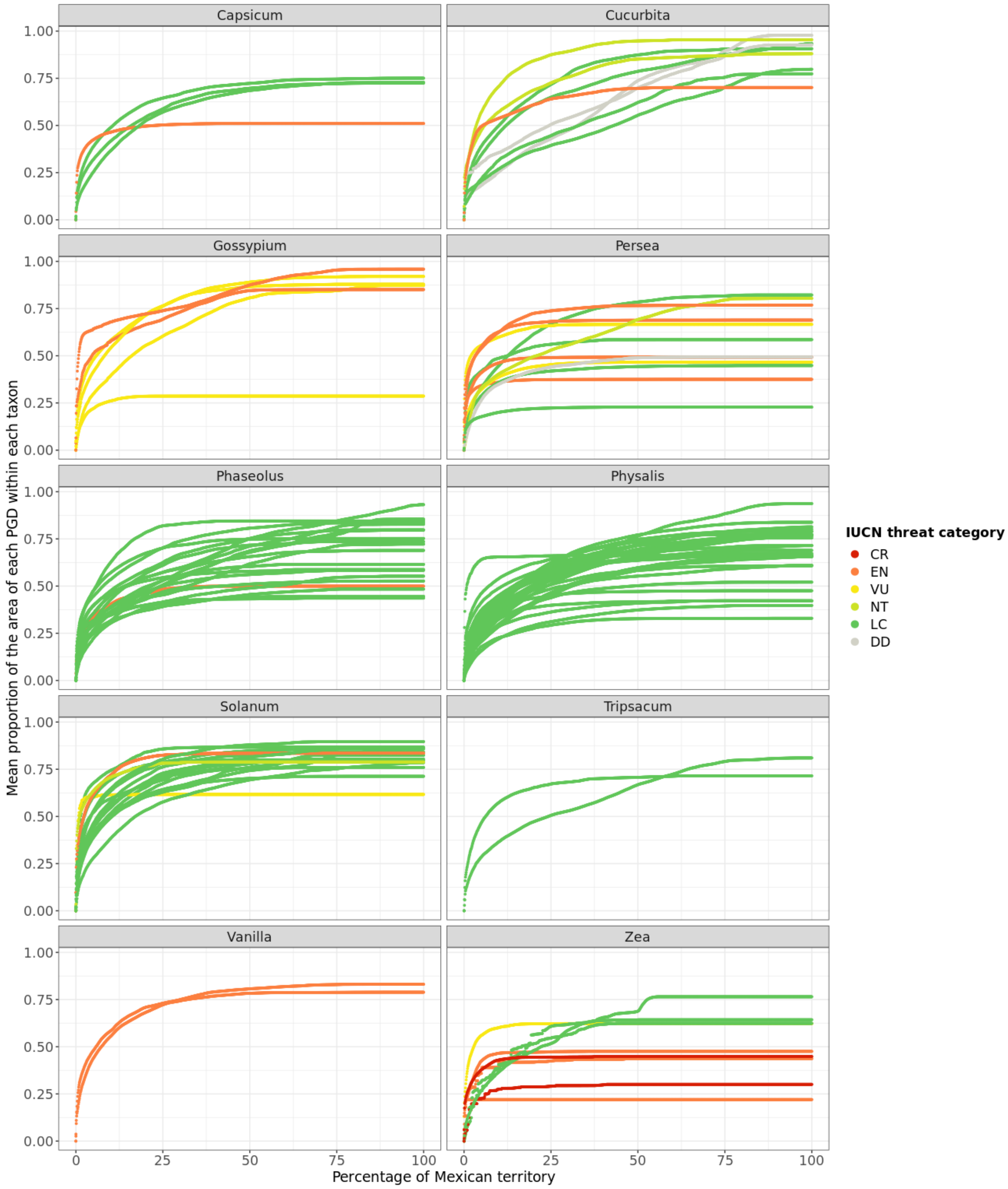

Supplementary Figure 10. Performance curves of how the proportion of taxa distribution ranges increased with the amount of land area. Taxa were grouped by genus. Colors are according to the IUCN Red List Category.

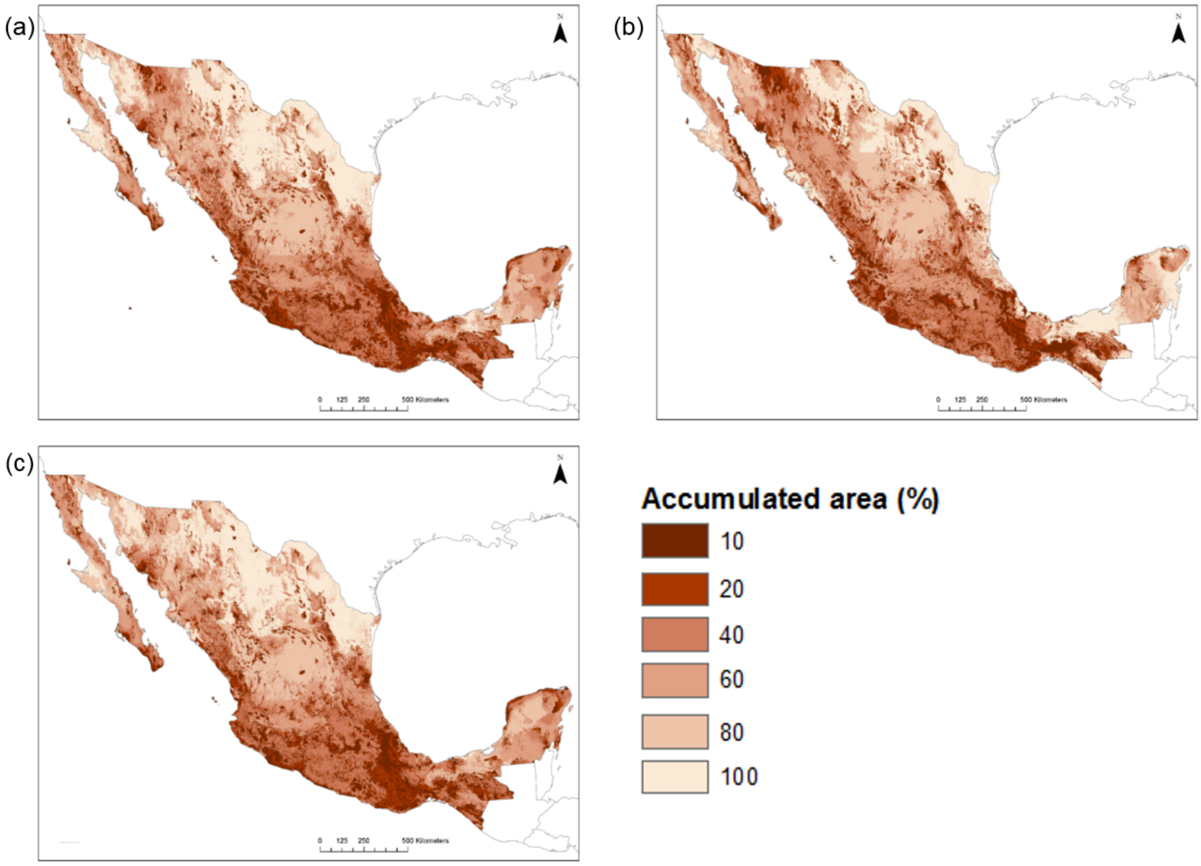

Supplementary Figure 11. Results of the systematic conservation planning process for Mesoamerican crop wild relatives in Mexico, considering (a) all taxa, (b) taxa exclusively distributing in natural vegetation, and (c) taxa associated with different habitats. The continuous hierarchical maps represent a ranking of the landscape where the 10% most valuable area is within the most valuable 20%; the most valuable 20% is within the most valuable 30%; thus expressed as accumulated area. [Country boundaries according to Natural Earth.]

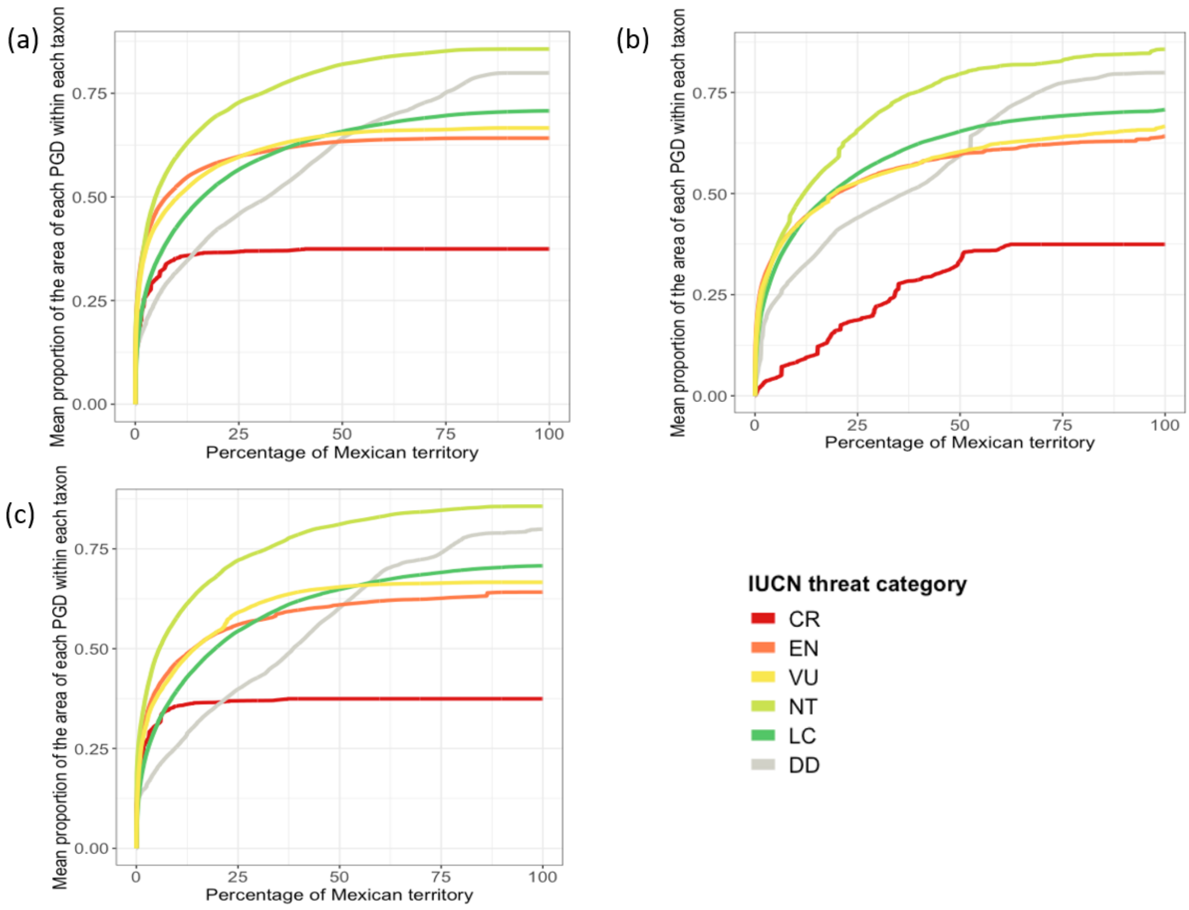

Supplementary Figure 12. Performance curves of all three scenarios quantifying how the proportion of taxa distribution ranges increased with the amount of land area (see maps at Supplementary Figure 11). Taxa were grouped by IUCN Red List Category. Scenarios considered (a) all taxa, (b) taxa exclusively distributing in natural vegetation, and (c) taxa associated with different habitats, *i.e.* natural vegetation, agricultural and urban areas.

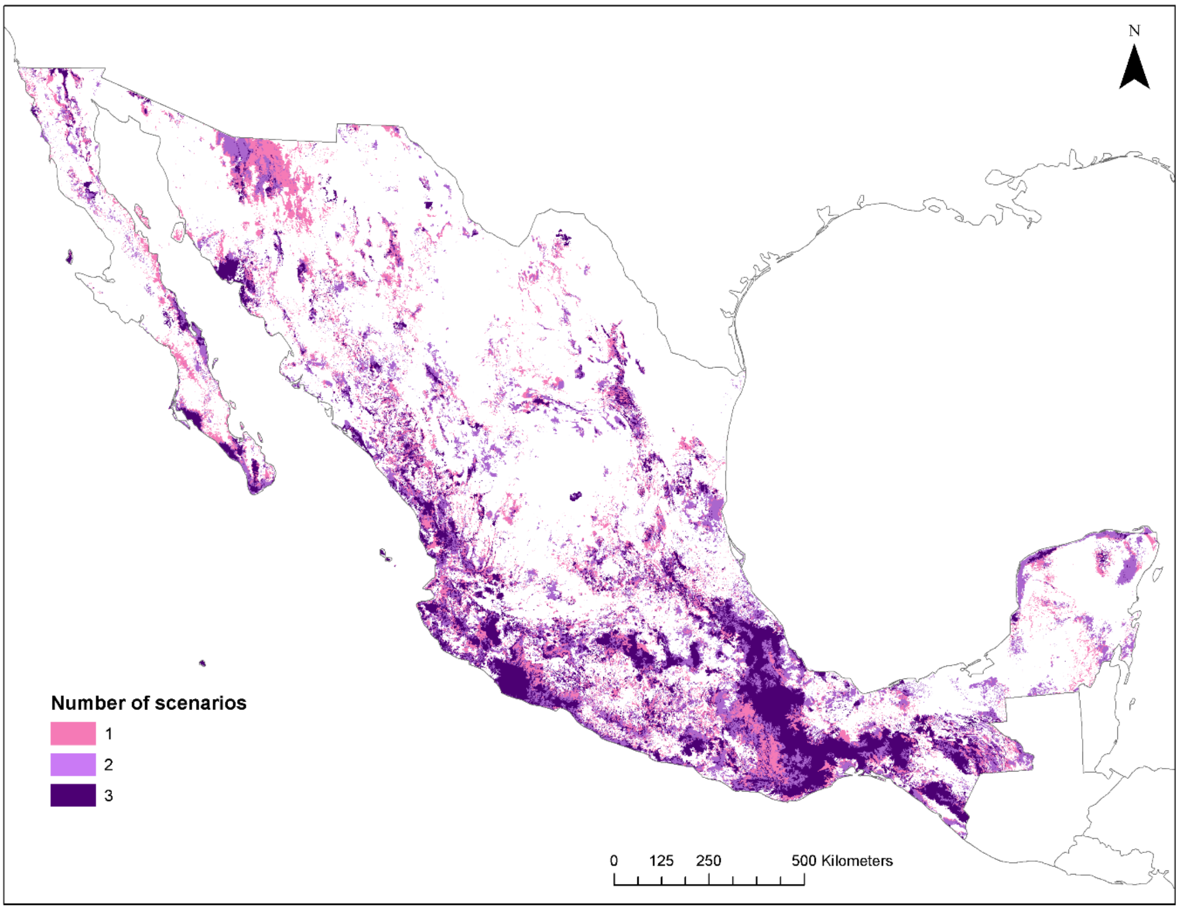

Supplementary Figure 13. Coincidence of three conservation scenarios, considering (a) all taxa, (b) taxa exclusively distributing in natural vegetation, and (c) taxa associated with different habitats. The most valuable 20% of Mexico's terrestrial area of each scenario was determined as a conservation area (extracted from the hierarchical maps, see Supplementary Figure 11). [Country boundaries according to Natural Earth.]

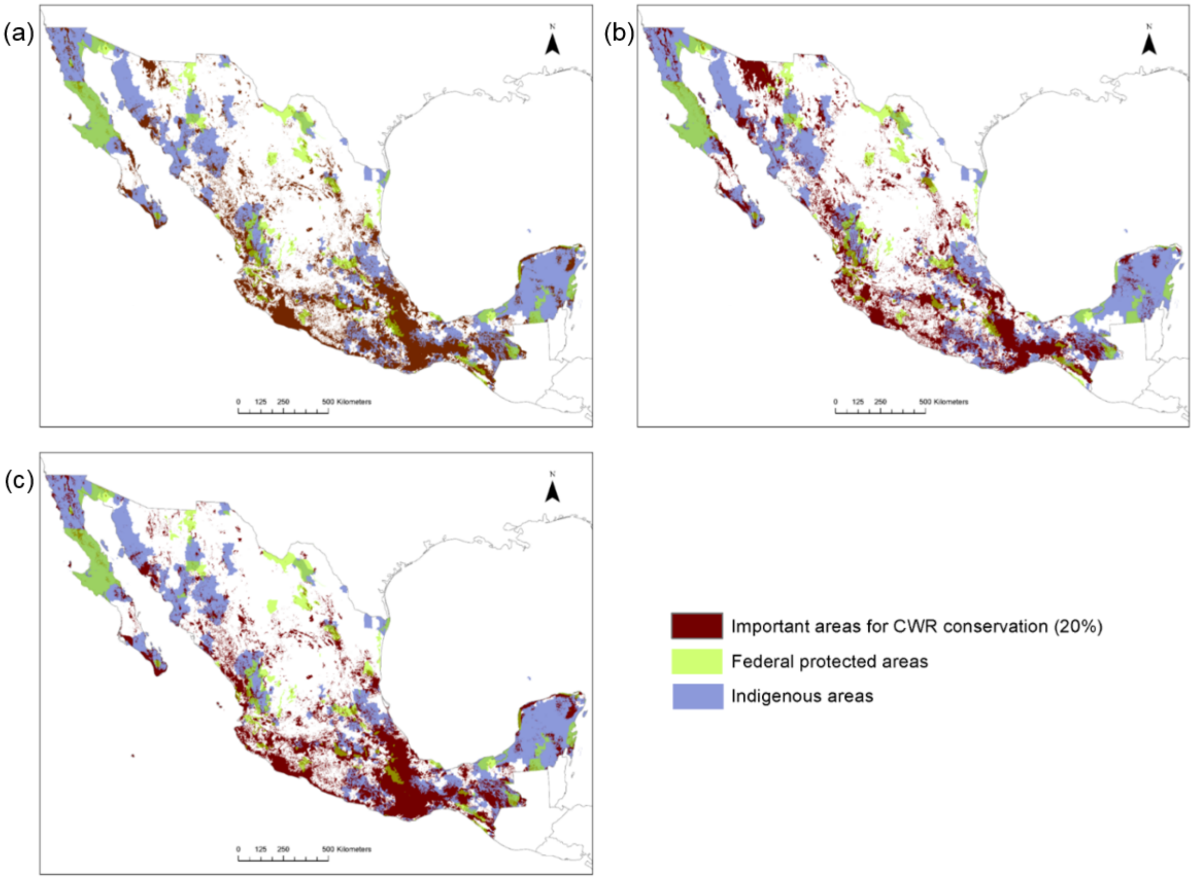

Supplementary Figure 14. Conservation area proposal for Mesoamerican crop wild relatives in Mexico, considering 20% of Mexico's terrestrial area that maximized the representation of taxa and PGD (see Supplementary Figures 11 and 12) and its coincidence with federal protected areas[69] and indigenous areas[70,71], although these criteria were not considered in the analysis. Scenarios included: (a) all taxa, (b) taxa exclusively distributing in natural vegetation, and (c) taxa associated with different habitats. [Spatial data is licensed under CC-BY 2.5; country boundaries according to Natural Earth.]

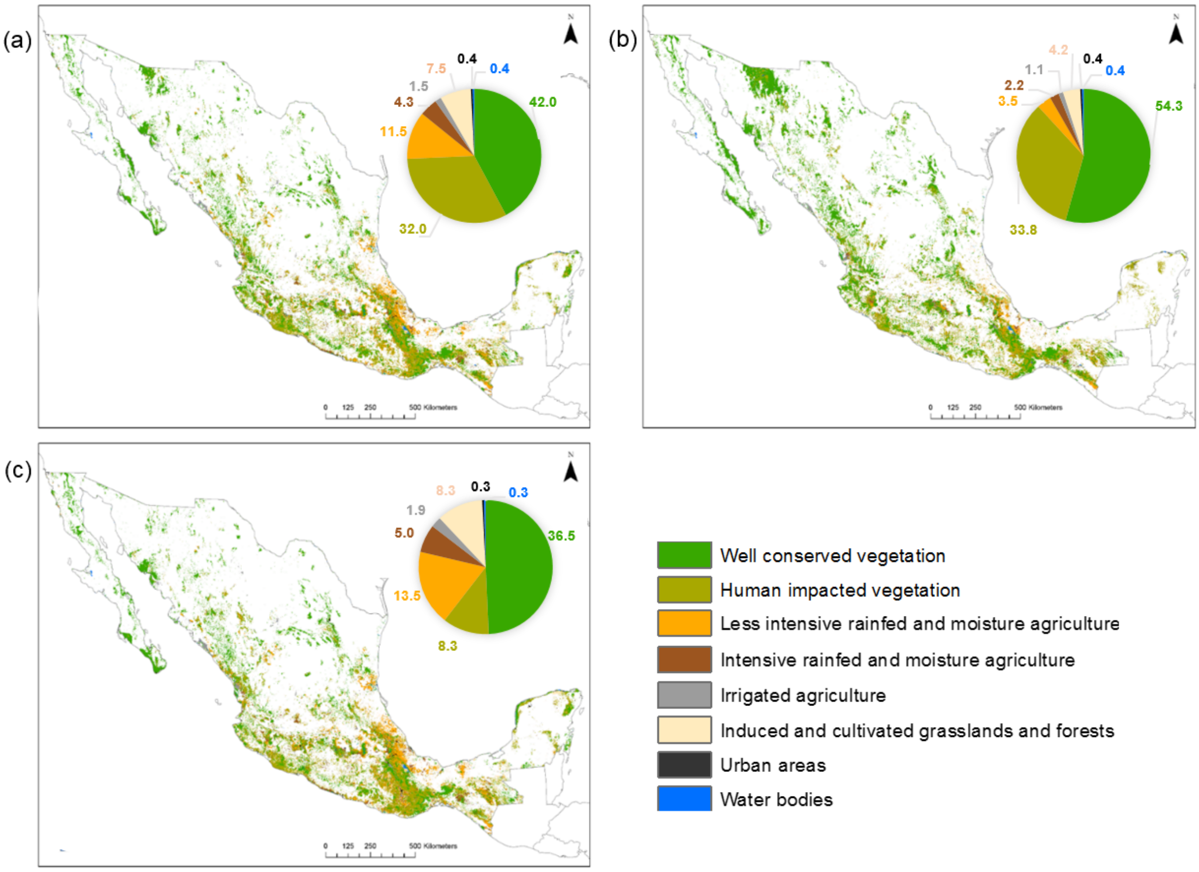

Supplementary Figure 15. Conservation area proposal for Mesoamerican crop wild relatives in Mexico considering 20% of Mexico's terrestrial area that maximized the representation of taxa and PGD (see Supplementary Figures 11 and 12) according to land cover data used for the analysis (see Supplementary Figure 9). Scenarios considered: (a) all taxa, (b) taxa exclusively distributing in natural vegetation, and (c) taxa associated with different habitats. [Spatial data is licensed under CC-BY 2.5; country boundaries according to Natural Earth.]

Supplementary Note 1.

Using the IUCN Red List Categories and Criteria, one-third (71 taxa) of the evaluated CWR are threatened (CR: 7 taxa., EN: 47 taxa, and VU: 16 taxa; Supplementary Data 3). Twenty taxa were assessed as DD as there was insufficient data to evaluate them. Cotton and vanilla were the most threatened groups with 92% and 89% of their evaluated species, respectively, at risk of extinction[72].

Threats affecting CWR include habitat loss and degradation —mainly due to the expansion of crops, livestock and infrastructure, such as urban development and roads, native pests, exotic invasive species and genetically modified organisms. Other main threat factors are climate change and extreme weather events. While the impact of these factors may be independent, in general, there are several stressors affecting (agro-)biodiversity, interacting in synergy and in complex ways, thus exacerbating their effects[73].

Supplementary Note 2.

The evaluation of the distribution of occurrence data in a 5 km^2 grid shows that areas of highest number of taxa are located in the central part of Mexico, particularly in the valley of Mexico, in the surroundings of Jalapa and in the region of Los Tuxtlas in the state of Veracruz, in the basin of Tehuacán-Cuicatlán, as well as in the surroundings of San Cristóbal de las Casas, Chiapas, in South Mexico (Supplementary Figure 1). Areas where taxa concentrate (i.e. 'high taxa richness' areas) are broadly similar to those based on SDM (Figure 2). Although sampling effort for each genus has not been assessed to disentangle its effects on taxon distribution pattern, it is likely that there is bias given that the main source of data is historical information from different herbaria and collection events. Location of research centers or biological stations might partly explain some of the areas with the highest number of CWR based on occurrence records.

Supplementary Note 3.

The protected areas with highest numbers of recorded taxa are CADNR 043 Estado de Nayarit (46 taxa), and Sierra de Manantlán (42 taxa); they also represent the protected areas with highest numbers of taxa based on SDM (Supplementary Data 7). However, most of the protected areas had considerable differences between the observed and estimated number of taxa, see for example Sierra de Quila (4 *vs.* 65 taxa, respectively), Insurgente José María Morelos (5 *vs.* 62 taxa, respectively), and Sierra de Huautla (6 *vs.* 60 taxa, respectively). Only nine protected areas indicate that approximately half of the estimated number of taxa have been recorded, including the protected areas of Revillagigedo, Bahía de Loreto, and El Pinacate y Gran Desierto de Altar. In these areas, on average, less than five taxa have been reported, but up to 11 taxa are potentially distributed in these areas. SDM represent sites with environmental conditions similar to where the species has been observed, i.e. presence data rather than absence information is used to obtain SDM, thus estimating the commission error is difficult[74].

Supplementary Note 4.

A major difference between the representation curves of the three scenarios is evident regarding the taxa that are critically endangered (Supplementary Figure 12). Most of them are distributed in different habitat types (Supplementary Data 15), so their representation was favored in the scenarios (a) that included all taxa and (c) that included taxa associated with different habitats and land uses (e.g. natural vegetation, agriculture and urban areas), but not in scenario (b) that focused on taxa exclusively distributed in natural vegetation.

Average values differ among groups with different risk of extinction due to the conservation weight. Although taxa with high risk of extinction (CR and EN) had highest conservation weights, which generally result in highest representation, here the results show a different pattern as their potential distribution ranges are highly impacted by anthropogenic factors, such as forest loss or degradation. Consequently, it was not possible to represent 100% of the potential distribution of taxa.

Supplementary Note 5.

Both the Gene Pool and Taxon Group (eg. same genus) concepts can be used to identify a wild relative of a crop[74]. The Taxon group concept allows to determine the degree of CWR relatedness based on taxonomic information. It assumes a positive correlation between taxonomic and genetic distance. Its application has been particularly useful to prioritize crops and its CWR for conservation actions.

Supplementary Note 6.

Zonation configuration

Settings file

[Settings]
removal rule = 1 (# Core Area Zonation)
warp factor = 1
edge removal = 1
annotate name = 0

use SSI = 1
SSI file name = SSI_list.txt

use groups = 1
groups file = habitat_group_features.txt

use condition layer = 1
condition file = habitat_features.txt

Batch file (run in cluster)

#!/bin/bash
zig4 -r E_final.dat features_Todos.spp /home/see/E_final/E_final_Todos.txt 0.0 0 0 0 (# analysis for all taxa)

#!/bin/bash
zig4 -r E_final.dat features_VegPyS.spp /home/see/E_final/E_final_VegPyS.txt 0.0 0 0 0 (# analysis for taxa exclusively distributed in well-preserved vegetation)

#!/bin/bash
zig4 -r E_final.dat features_HabVarios.spp /home/see/E_final/E_final_HabVarios.txt 0.0 0 0 0 (# analysis for taxa that can be associated to different habitats and land uses (e.g. natural vegetation, agriculture and urban areas)

Supplementary references

[1] Guevara, M. & Arroyo-Cruz, C. E. Modelo digital de elevaciones resolución 1 km. Escala: 1:4 000 000. Edición: 1. (Comisión Nacional para el Conocimiento y Uso de la Biodiversidad, 2016)

[2] CONABIO. Provincias biogeográficas de México. Escala 1:4 000 000 (Comisión Nacional para el Conocimiento y Uso de la Biodiversidad, 1997).

[3] Jaeger, J. R., Riddle, B. R. & Bradford, D. F. Cryptic Neogene vicariance and Quaternary dispersal of the red-spotted toad (*Bufo punctatus*): insights on the evolution of North American warm desert biotas. *Mol. Ecol.* **14**, 3033–3048 (2005).

[4] O'Connell, K. A., Streicher, J. W., Smith, E. N. & Fujita, M. K. Geographical features are the predominant driver of molecular diversification in widely distributed North American whipsnakes. *Mol. Ecol.* **26**, 5729–5751 (2017).

[5] Riddle, B. R. & Hafner, D. J. A step-wise approach to integrating phylogeographic and phylogenetic biogeographic perspectives on the history of a core North American warm deserts biota. *J. Arid Environ* **66**, 435–461 (2006).

[6] Wilson, J. S. & Pitts, J. P. Identifying Pleistocene refugia in North American cold deserts using phylogeographic analyses and ecological niche modelling. *Divers. Distrib* **18**, 1139–1152 (2012).

[7] Loera, I., Ickert-Bond, S. M. & Sosa, V. Pleistocene refugia in the Chihuahuan Desert: the phylogeographic and demographic history of the gymnosperm *Ephedra compacta. J. Biogeogr.* **44**, 2706–2716 (2017).

[8] Vásquez-Cruz, M. & Sosa, V. New insights on the origin of the woody flora of the Chihuahuan Desert: The case of Lindleya. *Am. J. Bot.* **103**, 1694–1707 (2016).

[9] Nason, J. D., Hamrick, J. L. & Fleming, T. H. Historical vicariance and postglacial colonization effects on the evolution of genetic structure in *Lophocereus*, a Sonoran Desert columnar cactus. *Evolution* **56**, 2214–2226 (2002).

[10] Riddle, B. Phylogeography and systematics of the *Peromyscus eremicus* species group and the historical biogeography of North American warm regional deserts. *Mol. Phylogenet. Evol.* **17**, 145–160 (2000).

[11] Rodríguez-Robles, J. A. & De Jesús-Escobar, J. M. Molecular systematics of new world gopher, bull, and pinesnakes (Pituophis: Colubridae), a transcontinental species complex. *Mol. Phylogenet. Evol.* **14**, 35–50 (2000).

[12] Bernardi, G. Baja California disjunctions and phylogeographic patterns in sympatric *California blennies. Front. Ecol. Evol.* **2**, 53 (2014).

[13] Stepien, C. A., Rosenblatt, R. H. & Bargmeyer, B. A. Phylogeography of the spotted sand bass, *Paralabrax maculatofasciatus*: divergence of Gulf of California and Pacific Coast populations. *Evolution* **55**, 1852–1862 (2001).

[14] González-Rubio, C., García-De León, F. J. & Rodríguez-Estrella, R. Phylogeography of endemic Xantus' hummingbird (*Hylocharis xantusii*) shows a different history of vicariance in the Baja California Peninsula. *Mol. Phylogenet. Evol.* **102**, 265–277 (2016).

[15] Mathis, V. L., Hafner, M. S. & Hafner, D. J. Evolution and phylogeography of the *Thomomys umbrinus* species complex (Rodentia: Geomyidae). *J. Mammal.* **95**, 754–771 (2014).

[16] Bryson, R. W., Murphy, R. W., Graham, M. R., Lathrop, A. & Lazcano, D. Ephemeral Pleistocene woodlands connect the dots for highland rattlesnakes of the *Crotalus intermedius* group. *J. Biogeogr.* **38**, 2299–2310 (2011).

[17] Bryson, R. W., García-Vázquez, U. O. & Riddle, B. R. Diversification in the Mexican horned lizard *Phrynosoma orbiculare* across a dynamic landscape. *Mol. Phylogenet. Evol.* **62**, 87–96 (2012).

[18] Bryson, R. W., García-Vázquez, U. O. & Riddle, B. R. Relative roles of Neogene vicariance and Quaternary climate change on the historical diversification of bunchgrass lizards (*Sceloporus scalaris* group) in Mexico. *Mol. Phylogenet. Evol.* **62**, 447–457 (2012).

[19] Bryson, R. W. & Riddle, B. R. Tracing the origins of widespread highland species: a case of Neogene diversification across the Mexican sierras in an endemic lizard. *Biol. J. Linn. Soc.* **105**, 382–394 (2011).

[20] Friis, G., Aleixandre, P., Rodríguez-Estrella, R., Navarro-Sigüenza, A. G. & Milá, B. Rapid postglacial diversification and long-term stasis within the songbird genus *Junco*: phylogeographic and phylogenomic evidence. *Mol. Ecol.* **25**, 6175–6195 (2016).

[21] Gugger, P. F., González-Rodríguez, A., Rodríguez-Correa, H., Sugita, S. & Cavender-Bares, J. Southward Pleistocene migration of Douglas-fir into Mexico: phylogeography, ecological niche modeling, and conservation of 'rear edge' populations. *New Phytol.* **189**, 1185–1199 (2011)

[22] Jaramillo-Correa, J. P., Beaulieu, J., Ledig, F. T. & Bousquet, J. Decoupled mitochondrial and chloroplast DNA population structure reveals Holocene collapse and population isolation in a threatened Mexican-endemic conifer. *Mol. Ecol.* **15**, 2787–2800 (2006).

[23] Jaramillo-Correa, J. P. et al. Ancestry and divergence of subtropical montane forest isolates: molecular biogeography of the genus *Abies* (Pinaceae) in southern México and Guatemala. *Mol. Ecol.* **17**, 2476–2490 (2008).

[24] Moreno-Letelier, A., Mastretta-Yanes, A. & Barraclough, T. G. Late Miocene lineage divergence and ecological differentiation of rare endemic *Juniperus blancoi*: clues for the diversification of North American conifers. *New Phytol.* **203**, 335–347 (2014).

[25] Moreno-Letelier, A. & Piñero, D. Phylogeographic structure of *Pinus strobiformis* Engelm. across the Chihuahuan Desert filter-barrier. *J. Biogeogr.* **36**, 121–131 (2009).

[26] Ortíz-Medrano, A., Moreno-Letelier, A. & Piñero, D. Fragmentación y expansión demográfica en las poblaciones mexicanas de *Pinus ayacahuite* var. *ayacahuite*. *Bol. Soc. Bot. México* **83**, 25–36 (2008).

[27] Parra-Olea, G., Windfield, J. C., Velo-Antón, G. & Zamudio, K. R. Isolation in habitat refugia promotes rapid diversification in a montane tropical salamander. *J. Biogeogr.* **39**, 353–370 (2012).

[28] Peñaloza-Ramírez, J. M., Rodríguez-Correa, H., González-Rodríguez, A., Rocha-Ramírez, V. & Oyama, K. High genetic diversity and stable Pleistocene distributional ranges in the widespread Mexican red oak *Quercus castanea* Née (1801) (Fagaceae). *Ecol. Evol.* **10**, 4204–4219 (2020).

[29] Rodríguez-Banderas, A., Vargas-Mendoza, C. F., Buonamici, A. & Vendramin, G. G. Genetic diversity and phylogeographic analysis of *Pinus leiophylla*: a post-glacial range expansion. *J. Biogeogr.* **36**, 1807–1820 (2009).

[30] Ruiz-Sanchez, E. & Specht, C. D. Ecological speciation in *Nolina parviflora* (Asparagaceae): lacking spatial connectivity along of the Trans-Mexican Volcanic Belt. *PLoS ONE* **9**, e98754 (2014).

[31] Wood, D. A., Vandergast, A. G., Lemos Espinal, J. A., Fisher, R. N. & Holycross, A. T. Refugial isolation and divergence in the narrowheaded gartersnake species complex (*Thamnophis rufipunctatus*) as revealed by multilocus DNA sequence data. *Mol. Ecol.* **20**, 3856–3878 (2011).

[32] Zhao, Y. et al. Comparative phylogeography of the *Smilax hispida* group (Smilacaceae) in eastern Asia and North America – Implications for allopatric speciation, causes of diversity disparity, and origins of temperate elements in Mexico. *Mol. Phylogenet. Evol.* **68**, 300–311 (2013).

[33] Quintero-Corrales, C. et al. Allopatric instead of parapatric divergence in an ectomycorrhizal fungus (*Laccaria trichodermophora*) in tropical sky-islands. *Fungal Ecol.* **47**, 100966 (2020).

[34] Andersen, J. J. & Light, J. E. Phylogeography and subspecies revision of the hispid pocket mouse, *Chaetodipus hispidus* (Rodentia: Heteromyidae). *J. Mammal.* **93**, 1195–1215 (2012).

[35] Rodríguez-Correa, H. et al. Complex phylogeographic patterns indicate Central American origin of two widespread Mesoamerican *Quercus* (Fagaceae) species. *Tree Genet. Genomes.* **13**, (2017).

[36] Smith, B. T. et al. The role of historical and contemporary processes on phylogeographic structure and genetic diversity in the Northern Cardinal, *Cardinalis cardinalis. BMC Evol. Biol.* **11**, 136 (2011).

[37] Zarza, E., Reynoso, V. H. & Emerson, B. C. Diversification in the northern neotropics: mitochondrial and nuclear DNA phylogeography of the iguana *Ctenosaura pectinata* and related species. *Mol. Ecol.* **17**, 3259–3275 (2008).

[38] Castillo-Chora, V. D. J., Sánchez-González, L. A., Mastretta-Yanes, A., Prieto-Torres, D. A. & Navarro-Sigüenza, A. G. Insights into the importance of areas of climatic stability in the evolution and maintenance of avian diversity in the Mesoamerican dry forests. *Biol. J. Linn. Soc.* **132**, 741–758 (2021).

[39] Hasbun, C. R., Gomez, A., Kohler, G. & Lunt, D. H. Mitochondrial DNA phylogeography of the Mesoamerican spiny-tailed lizards (*Ctenosaura quinquecarinata* complex): historical biogeography, species status and conservation. *Mol. Ecol.* **14**, 3095–3107 (2005).

[40] Suárez-Atilano, M., Burbrink, F. & Vázquez-Domínguez, E. Phylogeographical structure within Boa constrictor imperator across the lowlands and mountains of Central America and Mexico. *J. Biogeogr.* **41**, 2371–2384 (2014).

[41] Leaché, A. D., Palacios, J. A., Minin, V. N. & Bryson, R. W. Phylogeography of the Trans-Volcanic bunchgrass lizard (Sceloporus bicanthalis) across the highlands of south-eastern Mexico. *Biol. J. Linn. Soc.* **110**, 852–865 (2013).

[42] Mastretta-Yanes, A. et al. Gene duplication, population genomics, and species-level differentiation within a tropical mountain shrub. *Genome. Biol. Evol.* **6**, 2611–2624 (2014).

[43] Castillo-Chora, V. de J., Zamudio-Beltrán, L. E., Pozo, C. & Hernández-Baños, B. E. Phylogeography of *Habia fuscicauda* (Cardinalidae) indicates population isolation, genetic divergence and demographic changes during the Quaternary climate shifts in the Mesoamerican rainforest. *J Ornithol.* ***162,*** 961–976 (2021).

[44] Nigenda-Morales, S. F. et al. Phylogeographic and diversification patterns of the white-nosed coati (*Nasua narica*): Evidence for south-to-north colonization of North America. *Mol. Phylogenet. Evol.* **131**, 149–163 (2019).

[45] Nolasco-Soto, J., González-Astorga, J., Espinosa de los Monteros, A., Galante-Patiño, E. & Favila, M. E. Phylogeographic structure of *Canthon cyanellus* (Coleoptera: Scarabaeidae), a Neotropical dung beetle in the Mexican Transition Zone: Insights on its origin and the impacts of Pleistocene climatic fluctuations on population dynamics. *Mol. Phylogenet. Evol.* **109**, 180–190 (2017).

[46] Ortiz-Rodriguez, A. E. et al. Genetic differentiation among *Psittacanthus rhynchanthus* (Loranthaceae) populations: novel phylogeographic patterns in the Mesoamerican tropical lowlands. *Plant Syst. Evol.* **306**, 10 (2020).

[47] Rocha-Méndez, A., Sánchez-González, L. A., González, C. & Navarro-Sigüenza, A. G. The geography of evolutionary divergence in the highly endemic avifauna from the Sierra Madre del Sur, Mexico. *BMC Evol. Biol.* **19**, 237 (2019).

[48] Wüster, W. et al. Tracing an invasion: landbridges, refugia, and the phylogeography of the Neotropical rattlesnake (Serpentes: Viperidae: *Crotalus durissus*). *Mol. Ecol.* **14**, 1095–1108 (2005).

[49] Cavers, S., Navarro, C. & Lowe, A. J. Chloroplast DNA phylogeography reveals colonization history of a Neotropical tree, *Cedrela odorata* L., in Mesoamerica. *Mol. Ecol.* **12**, 1451–1460 (2003).

[50] Guevara-Chumacero, L. M. et al. Molecular phylogeography of *Pteronotus davyi* (Chiroptera: Mormoopidae) in Mexico. *J. Mamm.* **91**, 220–232 (2010).

[51] Montalvo-Fernández, G. et al. Impact of Late Pleistocene-Holocene climatic fluctuations on the phylogeographic structure and historical demographics of *Zamia prasina* (Cycadales: Zamiaceae). *Bot. Sci.* **97**, 588–608 (2019).

[52] Ramírez-Barrera, S. M., Velasco, J. A., Orozco-Téllez, T. M., Vázquez-López, A. M. & Hernández-Baños, B. E. What drives genetic and phenotypic divergence in the Red-crowned Ant tanager (*Habia rubica*, Aves: Cardinalidae), a polytypic species? *Ecol. Evol.* **9**, 12339–12352 (2019).

[53] Vázquez-Miranda, H., Navarro-Sigüenza, A. G. & Omland, K. E. Phylogeography of the Rufous-Naped Wren (Campylorhynchus rufinucha): Speciation and Hybridization in Mesoamerica. *The Auk* **126**, 765–778 (2009).

[54] Ruiz-García, M. et al. Phylogeography of the mantled howler monkey (*Alouatta palliata*; Atelidae, Primates) across its geographical range by means of mitochondrial genetic analyses and new Insights about the phylogeny of Alouatta. *Folia Primatol.* **88**, 421–454 (2017).

[55] Arbeláez-Cortés, E., Nyári, Á. S. & Navarro-Sigüenza, A. G. The differential effect of lowlands on the phylogeographic pattern of a Mesoamerican montane species (*Lepidocolaptes affinis*, Aves: Furnariidae). *Mol. Phylogenet. Evol.* **57**, 658–668 (2010).

[56] Ornelas, J. F. et al. Comparative phylogeographic analyses illustrate the complex evolutionary history of threatened cloud forests of Northern Mesoamerica. *PLoS ONE* **8**, e56283 (2013).

[57] Pérez Consuegra, S. G. & Vázquez-Domínguez, E. Mitochondrial diversification of the *Peromyscus mexicanus* species group in Nuclear Central America: biogeographic and taxonomic implications. *J. Zool. Syst. Evol. Res.* **53**, 300–311 (2015).

[58] Rodríguez-Gómez, F. & Ornelas, J. F. Genetic divergence of the Mesoamerican azure-crowned hummingbird (*Amazilia cyanocephala*, Trochilidae) across the Motagua-Polochic-Jocotán fault system. *J. Zool. Syst. Evol. Res.* **52**, 142–153 (2014).

[59] Daza, J. M., Castoe, T. A. & Parkinson, C. L. Using regional comparative phylogeographic data from snake lineages to infer historical processes in Middle America. *Ecography* **33**, 343–354 (2010)

[60] Ornelas, J. F. & González, C. Interglacial genetic diversification of *Moussonia deppeana* (Gesneriaceae), a hummingbird-pollinated, cloud forest shrub in northern Mesoamerica. *Mol. Ecol. 23*, 4119–4136 (2014).

[61] Mastretta-Yanes, A. et al. Long-term in situ persistence of biodiversity in tropical sky islands revealed by landscape genomics. *Mol. Ecol.* **27**, 432–448 (2018).

[62] Uscanga, A., López, H., Piñero, D., Emerson, B. C. & Mastretta-Yanes, A. Evaluating species origins within tropical sky-islands arthropod communities. *J. Biogeogr.* **48**, 2199-2210 (2021).

[63] CNA.Cuencas Hidrológicas. Escala 1:250 000. (Comisión Nacional del Agua, 1998).

[64] Guevara, M. & Arroyo-Cruz, C. E. Modelo digital de elevaciones resolución 1km. Escala: 1:4 000 000. edición: 1. (Comisión Nacional para el Conocimiento y Uso de la Biodiversidad, 2016).

[65] SEMARNAP, Subsecretaría de Recursos Naturales. Mapa de suelos dominantes de la República Mexicana. Escala 1:4 000 000. (SEMARNAP, 1998).

[66] Rivera-Rodríguez, D. M. *Estudio de la Diversidad y Estructura Genómica del Teocintle.* Doctoral Thesis, Advisor: Sánchez, J.J. (Universidad de Guadalajara, 2018).

[67] INEGI. Conjunto de Datos Vectoriales de Uso de Suelo y Vegetación. Escala 1:250 000. Serie VI (Capa Unión). (Instituto Nacional de Estadística y Geografía, 2016).

[68] Bellon, M. R. *et al.* Evolutionary and food supply implications of ongoing maize domestication by Mexican campesinos. *Proc. R. Soc. B Biol. Sci.* **285**, 20181049 (2019).

[69] CONANP. Áreas Naturales Protegidas Federales de México. Mayo 2017. Edición 1. Comisión Nacional de Áreas Naturales Protegidas. Ciudad de México, México. (2017).

[70] CONABIO. Población indígena en México por municipio, 2010. Escala 1:250 000. Comisión Nacional para el Conocimiento y Uso de la Biodiversidad. Datos estadísticos del 2010, del Instituto Nacional de Estadística y Geografía. México D.F. (2012).

[71] INPI. *Catálogo de localidades indígenas A y B 2020*. Secretaría de Bienestar, México (2019).

[72] Goettsch, B. et al. Extinction risk of Mesoamerican crop wild relatives. *Plants People Planet* ppp3.10225 (2021). doi:10.1002/ppp3.10225

[73] Anderson, R. P. Real *vs*. artefactual absences in species distributions: Tests for *Oryzomys albigularis* (Rodentia: Muridae) in Venezuela. *J. Biogeogr.* 30, 591–605 (2003).

[74] Maxted, N., Ford-Lloyd, B. V., Jury, S., Kell, S. & Scholten, M. Towards a definition of a crop wild relative. *Biodivers. Conserv.* 15, 2673–2685 (2006).
