# Supplementary material for: Incorporating evolutionary and threat processes into crop wild relatives conservation
Source: Nat Commun. 2022 Oct 21;13:6254. doi: 10.1038/s41467-022-33703-0 (PMC9587227; doi:10.1038/s41467-022-33703-0)
Supplement: Supplementary file 2 — Reporting Summary [file 41467_2022_33703_MOESM2_ESM.pdf]

nature portfolio

Corresponding author(s): Alicia Mastretta-Yanes

Last updated by author(s): Aug 22, 2022

Reporting Summary

Nature Portfolio wishes to improve the reproducibility of the work that we publish. This form provides structure for consistency and transparency in reporting. For further information on Nature Portfolio policies, see our Editorial Policies and the Editorial Policy Checklist.

Statistics

For all statistical analyses, confirm that the following items are present in the figure legend, table legend, main text, or Methods section.

n/a	Confirmed	
☐	☒	The exact sample size (*n*) for each experimental group/condition, given as a discrete number and unit of measurement
☒	☐	A statement on whether measurements were taken from distinct samples or whether the same sample was measured repeatedly
☒	☐	The statistical test(s) used AND whether they are one- or two-sided<br>*Only common tests should be described solely by name; describe more complex techniques in the Methods section.*
☒	☐	A description of all covariates tested
☒	☐	A description of any assumptions or corrections, such as tests of normality and adjustment for multiple comparisons
☐	☒	A full description of the statistical parameters including central tendency (e.g. means) or other basic estimates (e.g. regression coefficient) AND variation (e.g. standard deviation) or associated estimates of uncertainty (e.g. confidence intervals)
☒	☐	For null hypothesis testing, the test statistic (e.g. *F*, *t*, *r*) with confidence intervals, effect sizes, degrees of freedom and *P* value noted<br>*Give P values as exact values whenever suitable.*
☒	☐	For Bayesian analysis, information on the choice of priors and Markov chain Monte Carlo settings
☒	☐	For hierarchical and complex designs, identification of the appropriate level for tests and full reporting of outcomes
☒	☐	Estimates of effect sizes (e.g. Cohen's *d*, Pearson's *r*), indicating how they were calculated

Our web collection on statistics for biologists contains articles on many of the points above.

Software and code

Policy information about availability of computer code

Data collection	No software was used for data collection
Data analysis	Analyses were carried out in: Zonation version 4 and in R version 3.5.1 and 3.6.0. The following R packages were used: ENMeval_0.3.0, purrr_0.3.4, tidyr_1.0.2 dplyr_1.0.2 ggplot2_3.3.3, readr_1.4.0, gridExtra_2.3, ggnewscale_0.4.5, scatterpie_0.1.5, pophelper_2.3.1, rgdal_1.4-8, raster_3.4-5, sp_1.4-4, scales_1.2.0 , rgl_0.107.10, vegan_2.6-2 and pcadapt_4.3.3 . Species Distribution Models were done with Maxent 3.3.1. Collinearity between variables was assessed with the 'corselect' function of the package fuzzySim 1.0. Maps were plotted with ArcGIS 10.2.2.<br><br>Custom R scripts and Zonation files used for the analyses and figures of this study are available at the Github repository https://github.com/CONABIO/analisisUniCons_proxiGen (Mastretta-Yanes et al. 2022). Zonation settings are also available in Supplementary Note 5.<br><br>Mastretta-Yanes, A., et al (2022). Incorporating evolutionary and threat processes into crop wild relatives conservation. CONABIO/analisisUniCons_proxiGen: v.1.0.0. In Nature Communications (v.1.0). Zenodo. https://doi.org/10.5281/zenodo.7015703

For manuscripts utilizing custom algorithms or software that are central to the research but not yet described in published literature, software must be made available to editors and reviewers. We strongly encourage code deposition in a community repository (e.g. GitHub). See the Nature Portfolio guidelines for submitting code & software for further information.

Data

Policy information about availability of data

All manuscripts must include a data availability statement. This statement should provide the following information, where applicable:

- Accession codes, unique identifiers, or web links for publicly available datasets
- A description of any restrictions on data availability
- For clinical datasets or third party data, please ensure that the statement adheres to our policy

The Zonation input and output files, including potential species distribution models (SDM), occurrence records, table with IUCN category per taxa, habitat rasters, genetic data and metadata used in this study are available in Dryad under accession (https://doi.org/10.5061/dryad.7m0cfxpxm)123. SDM can also be downloaded at Conabio's GIS portal (http://www.conabio.gob.mx/informacion/gis/; and Supplementary Data 6 for direct download links). Source data to make Figures 3, 4 and 6 are provided with this paper.

To compile occurrence records, hundreds of data sources were consulted, including published and personal databases of the project participants, the Agrobiodiversity Atlas of Guatemala (https://www.ars.usda.gov/northeast-area/beltsville-md-barc/beltsville-agricultural-research-center/national-germplasm-resources-laboratory/docs/atlas-of-guatemalan-crop-wild-relatives), the Global Biodiversity Information Facility (GBIF, https://www.gbif.org/), and Mexico's Biodiversity Information System (SNIB, http://snib.mx/).

Field-specific reporting

Please select the one below that is the best fit for your research. If you are not sure, read the appropriate sections before making your selection.

☐ Life sciences ☐ Behavioural & social sciences ☒ Ecological, evolutionary & environmental sciences

For a reference copy of the document with all sections, see nature.com/documents/nr-reporting-summary-flat.pdf

Ecological, evolutionary & environmental sciences study design

All studies must disclose on these points even when the disclosure is negative.

Study description	We applied a modified version of a planning framework for CWR conservation. It included: (i) a CWR checklist, i.e. list of CWR taxa distributed in an area, (ii) a CWR inventory, i.e. taxa selection and collation of ancillary data, (iii) a taxa extinction risk assessment, and (iv) systematic conservation planning analyses for supporting in situ and ex situ conservation. The first three steps are detailed in Goettsch et al. (https://doi.org/10.1002/ppp3.10225). The novelty of our study relies on introducing a new approach in step (iv) to account for genetic differentiation in a spatially explicit way, through the use of proxies of genetic differentiation.
Research sample	We used the inventory of 224 (Supplementary Data 3) native or endemic taxa of Mesoamerican CWR (210 species and 14 subspecific taxa, including subspecies, races and varieties, Table 1). They are related to nine crops: chili pepper (Capsicum subsp.), squash (Cucurbita subsp.), cotton, (Gossypium subsp.), avocado (Persea subsp.), bean (Phaseolus subsp.), husk tomato (Physalis subsp.), potato (Solanum sect. Petota), maize (Zea subsp., and Tripsacum subsp.), and vanilla (Vanilla subsp.). These crops were chosen because they are native to Mesoamerica and were considered most relevant for the social, economic and biological characteristics of the region, identified during a stakeholder workshop. Additionally, the 224 taxa were evaluated according to the International Union for Conservation of Nature, IUCN, Red List Categories and Criteria.
Sampling strategy	A list of approximately 3000 CWR taxa (i.e. species, subspecies, varieties and subpopulations) belonging to the same genus of a crop cultivated or domesticated in Mesoamerica was compiled from different sources (e.g. Acevedo Gasman et al., 2009; Azurdia et al., 2011; Bellon et al., 2009; Perales & Aguirre, 2008). The list included 310 high priority CWR for Mexico (Contreras-Toledo et al., 2018), 105 taxa in Guatemala (Azurdia et al., 2011), 50 taxa in El Salvador (Chízmar-Fernández et al., 2009; Echeverría et al., 2008) and around 54 taxa in Honduras (Núñez & Alvarado, 1995). A subset of genera and their taxa (with the exception of Tripsacum, a tertiary gene pool relative of Zea mays) was selected for the present study following a set of criteria considered most relevant for the social, economic and biological characteristics of the region, identified during a stakeholder workshop. This selection is detailed in Goettsch et al. (https://doi.org/10.1002/ppp3.10225). From that list, we used the inventory of 224 native or endemic taxa that had enough data to asses their extinction risk. This dataset of 224 CWR is representative of the CWR of the main crops of Mesoamerica (10 genera). Since there is no minimun or maximun number of species for undertaking conservation planning analyses with Zonation, we used this taxa dataset (n=116 with SDM and n=98 with occurrence points).
Data collection	We integrated more than 13,000 curated occurrence records from published and personal databases of project participants, the Agrobiodiversity Atlas of Guatemala, the Global Biodiversity Information Facility (GBIF, https://www.gbif.org/), and Mexico's Biodiversity Information System (SNIB, http://snib.mx/). Data was curated by authors WT-N, BG, APC-R, EU-H, MAO-R and OO-G
Timing and spatial scale	We limited SDM and all spatial data to Mexico's borders. Data collection was done during 2017-2019 as part of the workshops of the "Safeguarding Mesoamerican Crop Wild Relatives" Darwin Initiative Project. Two records were updated in 2020 and 2021 as an error was found by experts on the taxa (co-authors of this study).
Data exclusions	We excluded taxa with insufficient data to perform analyses, as detailed in Goettsch et al. (https://doi.org/10.1002/ppp3.10225).
Reproducibility	All code was documented and commited at https://github.com/CONABIO/analisisUniCons_proxiGen, a release version was submitted to Zenodo https://doi.org/10.5281/zenodo.7015703. Data used by those scripts was deposited in a Dryad repository https://

	doi.org/10.5061/dryad.7m0cfxpxm. Data (as presented in the repository) was copied to independent servers and downstream analyses were re-run by AM-Y and WT-N independently. R html notebooks of plotting scripts are provided.
Randomization	Experts were assigned to taxon groups based on their own expertise to incorporate their knowledge on each taxon, group discussions and validation of SDM, making randomization not possible.
Blinding	Our study incorporated experts' knowledge on each taxon, group discussions and validation of SDM, making blinding not possible.

Did the study involve field work? ☐ Yes ☒ No

Reporting for specific materials, systems and methods

We require information from authors about some types of materials, experimental systems and methods used in many studies. Here, indicate whether each material, system or method listed is relevant to your study. If you are not sure if a list item applies to your research, read the appropriate section before selecting a response.

Materials & experimental systems

n/a	Involved in the study	
☒	☐	Antibodies
☒	☐	Eukaryotic cell lines
☒	☐	Palaeontology and archaeology
☒	☐	Animals and other organisms
☒	☐	Human research participants
☒	☐	Clinical data
☒	☐	Dual use research of concern

Methods

n/a	Involved in the study	
☒	☐	ChIP-seq
☒	☐	Flow cytometry
☒	☐	MRI-based neuroimaging
