# Supplementary material for: Incorporating evolutionary and threat processes into crop wild relatives conservation
Source: Nat Commun. 2022 Oct 21;13:6254. doi: 10.1038/s41467-022-33703-0 (PMC9587227; doi:10.1038/s41467-022-33703-0)
Supplement: Supplementary file 3 — Description of Additional Supplementary Files [file 41467_2022_33703_MOESM3_ESM.pdf]

Title: Supplementary Data 1.
Description: Crop wild relatives (CWR) checklist, including a list of almost 3,000 CWR taxa distributed in Mesoamerica.

Title: Supplementary Data 2.
Description: CWR subset, including a list of more than 500 CWR taxa as a result of a first round to subset the CWR checklist.

Title: Supplementary Data 3.
Description: CWR inventory of selected Mesoamerican CWR, IUCN Red List Criteria and Category (see details in Goettsch *et al.* 2021), and the spatial format used for analysis (SDM: species distribution model; Occs: occurrence georeferenced data). (IUCN Red List Category, CR: Critically endangered, EN: Endangered, VU: Vulnerable, NT: Near threatened, LC: Least concern, DD: Data deficiency). The inventory included the list of 224 CWR taxa resulting from the final subsetting of the CWR checklist which were used for the present assessment.

Title: Supplementary Data 4.
Description: CWR occurrence records used to obtain potential species distribution models.

Title: Supplementary Data 5.
Description: MaxEnt performance and significance of potential species distribution models. (Occurrence data was divided into 70% for training and 30% for testing the model. MaxEnt performance was tested with 30% of the withheld data. Testing omission rate is given for SDM selected by experts, which were mainly model threshold with the ten percentile training presence.) (*indicates omission rates under a minimum training presence threshold and ✛ indicates SDM with AUC values below 0.75).

Title: Supplementary Data 6.
Description: References of the species distribution models of Mesoamerican CWR.

Title: Supplementary Data 7.
Description: Information of CWR in Mexican protected areas. Data is based on taxa occurrences (occs.) of and potential species distribution models (SDM).

Supplementary Data 8.
Description: Holdridge life zones characterization of Mexico, that integrates data on bio-temperature, annual precipitation and potential evapotranspiration ratio, used as environmental information to assess proxies of genetic differentiation.

Title: Supplementary Data 9.
Description: References used to subdivide the life zones into proxies of genetic differentiation.

Title: Supplementary Data 10.
Description: Taxonomic groups used in studies that were used to assess phylogeographic patterns in Mexico. (See references at Supplementary Data 9.)

Title: Supplementary Data 11.
Description: Habitat preferences of Mesoamerican CWR with potential species distribution model used in the spatial analysis (1: high preference; 0.5: low preference; 0.1: no preference).

Title: Supplementary Data 12.
Description: Area of the three conservation scenarios of Mesoamerican CWR in federal protected areas and indigenous areas of Mexico. We assessed 20% of Mexico's terrestrial area as a conservation area in order to maximize the representation of taxa and proxies of genetic differentiation.
